# Moving beyond Weight: A Narrative Review of the Dietary and Lifestyle Management for Reducing Cardiometabolic Risk in Polycystic Ovary Syndrome (PCOS)

**DOI:** 10.3390/nu15245069

**Published:** 2023-12-11

**Authors:** Shannon Herbert, Kathleen Woolf

**Affiliations:** Department of Nutrition and Food Studies, Steinhart School of Culture, Education, and Human Development, New York University, New York, NY 10003, USA; slh465@nyu.edu

**Keywords:** polycystic ovary syndrome, cardiometabolic risk, body weight, weight management, dietary patterns

## Abstract

Polycystic ovary syndrome (PCOS) is the most common endocrine disorder experienced by women. PCOS is a lifelong condition associated with reproductive, metabolic, and psychological presentations. PCOS is also linked with increased prevalence of cardiometabolic risk factors. While an association between body weight and PCOS has been noted, cardiometabolic risk factors are prevalent in individuals with PCOS across body weights. Currently, no consensus exists as to the most appropriate lifestyle strategy for mitigating cardiometabolic risk in PCOS. A large proportion of the literature is focused on weight loss for individuals with PCOS who are overweight or experience obesity, despite PCOS being prevalent across body sizes. The aim of this narrative review is to assess dietary and lifestyle interventions aimed at reducing cardiometabolic risk in individuals with PCOS across body sizes. A total of 51 articles are included in this review. Overall, randomized controlled trials are limited and most studies focus on weight loss, excluding individuals classified within a healthy body weight range. Studies that modified the dietary pattern without an energy deficit saw improvements in cardiometabolic risk. Thus, less restrictive dietary approaches may be effective at reducing cardiometabolic risk in this population. This review also highlights the need for more sustainable lifestyle interventions that meet the needs of individuals with PCOS of varying body weights.

## 1. Introduction

Polycystic ovary syndrome (PCOS) is the most common endocrine disorder experienced by women, affecting up to 20% of reproductive age women worldwide [1,2]. PCOS is characterized and diagnosed by the presence of menstrual irregularities, hyperandrogenism, and polycystic ovaries [2]. The symptoms experienced by individuals with PCOS vary but can include infertility; hirsutism, or excessive hair growth in areas males tend to have hair; thinning hair; weight gain or difficulty losing weight; acne or oily skin; and acanthosis nigricans, a thickening of dark patches on the skin [2]. PCOS is a lifelong condition associated with reproductive, metabolic, and psychological presentations [1,3,4].

PCOS is the most common cause of anovulatory infertility, presenting in approximately 75% of individuals with PCOS [5,6]. With such a large prevalence of infertility within the PCOS population, much of the literature has discussed strategies to improve reproductive outcomes [5,7]. Historically, PCOS has been thought solely as a reproductive disorder associated with infertility [8] and pregnancy complications [9]. However, the current understanding has shifted to recognize PCOS as a lifelong condition associated with several comorbidities.

PCOS is linked with increased prevalence of cardiometabolic risk factors, including dyslipidemia [10,11,12,13], hypertension [13,14,15], chronic inflammation, and impaired glucose tolerance [16,17], as well as overweight and obesity [18,19]. Individuals with PCOS have been shown to have higher body weight [20], fat mass [21], body fat percentage [10,22], body mass index (BMI) [14,20,23,24], waist circumference (WC) [10,20], and waist-to-hip ratio (WHR) [10,11,15,20,23,24,25] compared to individuals without PCOS. For instance, a recent systematic review and meta-analysis estimated the pooled prevalence of overweight and obesity among individuals with PCOS to be 61% (95% CI: 54–68%) and 49% (95% CI: 42–55%), respectively [18].

While an association between body weight and PCOS has been noted [18], cardiometabolic risk factors are prevalent in individuals with PCOS across varying body weights [19,26], implying that PCOS may be a significant risk factor for cardiovascular disease and type 2 diabetes, regardless of weight and BMI [19]. Insulin resistance appears to be a large driver of these risk factors, with nearly 75% of individuals with PCOS estimated to have some degree of insulin resistance [27]. Individuals with PCOS have presented with significantly higher glucose concentrations [21], post-load glucose [10,20,28], insulin concentrations [10,15,20,21,22,24,29,30], post-load insulin [10,25,31], and insulin resistance [10,14,20,21,29] than individuals without PCOS. Additionally, the homeostatic model assessment for insulin resistance (HOMA-IR), a marker of insulin resistance, has been positively associated with BMI [10,20,23,29] among individuals with PCOS.

Having a higher BMI appears to exacerbate insulin resistance by approximately 15% in individuals with PCOS [32]. Yet the correlation between BMI and insulin resistance has also been observed in controls without PCOS [10,29], suggesting that BMI is likely not the sole contributor to the higher prevalence of cardiometabolic risk factors observed in individuals with PCOS. Furthermore, observational studies that included BMI-matched controls still identified a higher prevalence of cardiometabolic risk factors in individuals with PCOS, irrespective of BMI [10,21,28,29,30,31]. Additionally, observational studies that have included individuals with PCOS with a healthy BMI (18.5–24.9 kg/m^2^) still identified a higher prevalence of cardiometabolic risk factors between individuals with and without PCOS [10,11,21,22,23,25,28,30,31,33]. This suggests that weight and BMI alone likely do not explain the prevalence of cardiometabolic risk factors observed in the PCOS population. The level of central adiposity likely plays a key role in cardiometabolic risk, as insulin resistance has been associated with higher levels of central adiposity in the PCOS population [32,34,35,36].

As the research literature demonstrates that dietary intake is a modifiable risk factor for cardiometabolic risk [37,38], the dietary intakes of individuals with and without PCOS have been explored. Despite individuals with PCOS having a higher prevalence of cardiometabolic risk factors, a systematic review and meta-analysis of 39,471 women with PCOS found that individuals with PCOS overall had comparable total energy, carbohydrate, fat, and protein intakes than individuals without PCOS [39]. Thus, energy intake alone may not be able to explain the higher prevalence of cardiometabolic risk in individuals with PCOS.

Yet observational studies suggest that the dietary intakes for individuals with PCOS may be associated with disease severity [14,21,40], including inflammatory status [21], insulin resistance [21,40], hirsutism [40], and cardiovascular health [14]. Lower intakes of key nutrients associated with health, such as complex carbohydrates [21], fiber [11,21,23,25,28], unsaturated fatty acids [21], omega-3 polyunsaturated fatty acids [15,21], vitamin A, folate, vitamin C [11], vitamin D, and magnesium [39], have been reported for individuals with PCOS compared to individuals without. Moreover, higher intakes of nutrients associated with poorer health outcomes, such as simple carbohydrates [21] and saturated fatty acids, have been reported for individuals with PCOS [21]. Furthermore, individuals with PCOS have been found to have a larger intake of foods high in salt, sugar, fat, and energy, with little to no protein, vitamins or minerals [25]. Furthermore, a higher adherence to an anti-inflammatory dietary pattern has been linked to lower PCOS risk [11,39,41]. Overall, women with PCOS have either poorer or comparable intakes of major food groups, including grains, fruits, vegetables, proteins, seeds, nuts, and dairy [39], as well as micronutrients, calcium, zinc, iron, folic acid, and vitamin D [39], suggesting room for improvement in the overall dietary patterns of individuals with PCOS.

While a healthy lifestyle is vital for the management of PCOS, currently, no consensus exists as to the most appropriate lifestyle strategy for managing PCOS. A large proportion of the literature is focused on weight loss for individuals with PCOS [42,43,44,45], despite PCOS being prevalent across body sizes [18,46]. Similarly, the literature tends to only include overweight or obese individuals as study participants [18,39,43,47]. Yet the overall relationship with PCOS and obesity is quite complex [4]. With a large proportion of research being conducted in medical centers [4,18,39], individuals at higher body weights may present with worse clinical presentations and perhaps are more likely to be diagnosed [18], referred to other providers [4,48,49], and ultimately included in the research. When comparing the prevalence of obesity in individuals with PCOS in medically unbiased or unreferred populations, the BMI distribution between individuals with and without PCOS appears to be more similar [4,48,49]. Thus, the overall understanding of PCOS management among individuals with different BMIs is greatly limited.

Additionally, individuals with PCOS have higher psychological concerns, including body image distress and disordered eating [1,50,51,52,53,54], making weight-focused interventions potentially inappropriate. Furthermore, weight loss interventions may not be sustainable and potentially lead to greater risk in the long term [55,56,57,58,59]. Lastly, there are benefits to a healthy lifestyle even in the absence of weight loss [1]. Thus, understanding lifestyle approaches that improve cardiometabolic risk but perhaps are not solely focused on weight loss are needed for this population to understand future directions for the nutritional management of PCOS. The 2023 International Evidence-Based Guidelines for the Assessment and Management of PCOS recommends that healthy lifestyle behaviors should be recommended to all women with PCOS and should be tailored to allow for a flexible, individual approach that avoids unduly restrictive or nutritionally unbalanced diets [1]. Thus, the aim of this narrative review is to assess the dietary and lifestyle interventions aimed at reducing cardiometabolic risk in individuals with PCOS across body sizes.

## 2. Methods

A literature search was conducted between June 2021 and April 2023 using PubMed and CINAHL to identify intervention studies that examined the relationship between lifestyle and cardiometabolic risk factors in individuals with PCOS. PubMed MeSH search terms with Boolean functions included “polycystic ovary syndrome” AND “diet” OR “exercise” AND “heart disease risk factors” OR “glucose metabolism disorders.” The search was limited to original human subjects research published in English between 1989 and 2023. Intervention studies were included if they targeted adult individuals with PCOS across weight and BMI ranges. Furthermore, selected studies must have included at least one anthropometric measure (body weight, BMI, WC, or body composition), as well as one biochemical (lipid profile and insulin or glucose concentrations) or clinical (blood pressure) marker in their outcomes. By including studies that measured at least one biochemical or clinical marker in addition to one anthropometric measure, we identified studies that looked at measures of cardiometabolic risk other than solely body weight, as our purpose was to review the literature on lifestyle interventions to mitigate cardiometabolic risk for individuals with PCOS across body sizes.

Studies were excluded if they were not published in English, examined adolescents, or did not contain at least one anthropometric measure as well as either a biochemical or clinical marker. Additionally, studies were excluded if the primary intervention included a vitamin, mineral, herbal supplement, or medication. Studies were categorized and organized into solely dietary interventions or lifestyle interventions. Dietary interventions were defined as interventions that modified different components of the diet, including energy intake, macronutrient composition, dietary pattern, or eating behavior, whereas lifestyle interventions modified the diet (as defined above) and included an additional behavioral modification (e.g., exercise or cognition). Dietary intervention studies were organized by the component of the diet altered. Lifestyle intervention studies were organized by the type of dietary and lifestyle behavior that was modified.

## 3. Results

A total of 51 studies (37 dietary intervention, 14 lifestyle intervention) were included in this review. The dietary strategies utilized varied widely, spanning from dietary changes in the form of energy restrictions to modifications of the dietary patterns without energy restriction. The additional behavioral modifications of the lifestyle varied widely and included structured and unstructured exercise, as well as cognitive behavior therapy interventions. There were a variety of diagnostic criteria used to diagnosis PCOS across studies, including Rotterdam, the 1990 National Institute of Health, and Androgen Excess Society criteria. A large majority of the studies diagnosed PCOS utilizing the Rotterdam criteria (55%). The studies included in the review are discussed below.

### 3.1. Dietary Interventions

As summarized in Table 1, the studies have examined the impact of dietary interventions on cardiometabolic risk factors in individuals with PCOS. These dietary interventions include changes in energy restriction, macronutrient distribution, dietary pattern, eating behavior, energy restriction and macronutrient distribution, and energy restriction and dietary pattern.

#### 3.1.1. Energy-Restricted Diets

Very Low-Calorie Diets.

Very low-calorie diets (VLCDs) (Table 1) comprising of approximately 450–800 calories per day have been studied as a mechanism to improve cardiometabolic risk in individuals with PCOS with overweight and obesity. While all of the included studies found VLCDs effective for weight loss [60,61,62,63,64,65], many of the studies are over 20 years old [60,61,63,65], lack a control group [61,62,63,64,65], do not describe the demographic characteristics of participants [60,61,62,63,64,65], and have relatively small sample sizes [60,61,62,63,65], limiting the generalizability of the interventions.

Short-term studies have shown that after following a VLCD, individuals with PCOS had lower body weight [60,61,62], and improved glucose [62] and insulin measures [60,61,62]. Longer term studies, consisting of a VLCD followed by a low-calorie diet for a period of six to seven months, have also observed decreases in body weight and insulin measures in participants with PCOS [63,64,65]. Yet the initial changes from following the VLCD may not be sustainable. Despite participants still following a low-calorie diet, the weight loss and decreases in total cholesterol and triglyceride concentrations from baseline were no longer significant six to seven months later [64,65]. Thus, the improvements in cardiometabolic risk factors from a VLCD may not be sustainable, despite continuing to follow a low-calorie diet.

Other Energy-Restricted Diets.

Energy-restricted diets, ranging from 500–1000 kcal/day energy deficits, have also been studied for individuals with PCOS with overweight and obesity (Table 1). Studies that explored energy restriction included replacing two meals daily with a meal replacement beverage [68], a 12-week 1000 kcal/day caloric deficit [70], and a 12-week 500 kcal/day deficit [71]. At the end of the meal replacement beverage intervention, significantly more PCOS participants exhibited insulin resistance post-intervention [68], implying weight loss itself may not always improve insulin resistance. While the researchers stated that reductions in insulin resistance might rely on reduction in a certain level of abdominal fat, there were no significant differences in total fat mass nor total fat-free mass markers between PCOS and non-PCOS participants in the study [68].

With a 1000 kcal/day deficit intervention, researchers found significant decreases in weight, WC, and fasting insulin [70]. Yet other researchers found that a 500 kcal/d deficit was also sufficient for an average weight loss of 3.5% that was significantly associated with a reduction in fasting blood glucose, plasma insulin, and LDL-C concentrations, as well as HOMA-IR [71]. Thus, a smaller, and perhaps more realistic, energy deficit has cardiometabolic benefits comparable with larger energy deficits.

Overall, these studies indicate that an energy-restricted diet may be beneficial at improving cardiometabolic risk factors in individuals with PCOS with overweight and obesity. Yet these studies are limited in their generalizability due to the lack of a detailed description of the intervention [67], racial and ethnic diversity of participants [68], discussion of the demographic characteristics [70], and control groups [70,71], as well as short study duration. The cardiometabolic risk factors measured differ across studies, further challenging a comparison of results. Furthermore, these energy-restrictive diets are likely not appropriate for individuals with PCOS within a healthy BMI range.

#### 3.1.2. Macronutrient Distribution

Low-Carbohydrate (CHO) Diet.

Other studies (Table 1) have looked to moderate or reduce the number of carbohydrates consumed to help mitigate cardiometabolic risk factors in individuals with PCOS across BMI ranges. The definition of low CHO varies across the studies included in this review, ranging from 40–43% of energy coming from CHO. These percentages of energy from CHO are not much lower than the Dietary Reference Intakes Acceptable Macronutrient Distribution Range (AMDR) for CHO, 45–65%, perhaps making these interventions more aptly described as moderate CHO rather than low CHO. All of the studies were designed to be eucaloric and found a low-CHO diet to lower fasting insulin [72,73,74], glucose [73], total cholesterol [72,73], LDL-C concentrations [73,74], HOMA-IR [73], and area under the curve insulin [74], as well as to improve insulin sensitivity [73] in individuals with PCOS.

The strengths of these studies include the provision of food for the study participants [72,73,74], randomization and blinding [73], and diverse samples [73]. Yet the limitations included small sample sizes [72,74] and short intervention periods [72,74]. Furthermore, researchers in one study purposively selected participants with overweight and obesity, stating that the findings would be more applicable to the general population [72]. Yet PCOS does affect individuals with different body sizes [18,46]. This purposive sampling scheme may limit the generalizability of the findings, despite this study indicating that weight loss was not required to see a reduction in cardiometabolic risk factors. Overall, these studies indicate again that even in the absence of weight loss, a modification of the dietary pattern may improve the presence of cardiometabolic risk factors in women with PCOS.

Protein Modification.

Rather than modifying the CHO content of the diet, the studies (Table 1) modified the protein intake to improve cardiometabolic risk profiles of individuals with PCOS across BMI categories. Two studies were included in this review, with one reducing the intake of animal protein while increasing intake of textured soy protein [75] and the other examining a high-protein diet (>40% of energy intake) [76]. While both studies improved markers of cardiometabolic risk [75,76], the limitations should be considered. In the textured soy protein study, all participants were prescribed metformin, regardless of intervention group. Thus, metformin may have influenced the improvements in cardiometabolic risk [75]. In the 6-month ad libitum high-protein study, there was a 53% attrition rate [76], which may have influenced the results and indicates that the intervention is not sustainable for this population. Though, overall, both of these studies again highlight that dietary modifications, irrespective of changes in energy, may promote improvement in cardiometabolic risk factors. While both studies studied protein modification, the interventions varied greatly, making it hard to come to any overall conclusions regarding the role of dietary protein to reduce cardiometabolic risk factors in individuals with PCOS. Nonetheless, modification of protein intake, regardless of BMI, may improve cardiometabolic risk factors in individuals with PCOS.

High-Saturated Fat, Starch Avoidant Diet.

In a unique study design, researchers [77] examined the effect of a high saturated fat, starch avoidant diet (HSF-SA) in individuals with PCOS with obesity (Table 1). Individuals were instructed to consume half of their total energy as saturated fat with restricted amounts of non-starchy vegetables and fruits. After 24 weeks, BMI, percent total body weight, and insulin concentrations were significantly reduced, with no differences in lipid profiles. Although the researchers suggested that a HSF-SA diet can lead to weight loss without adverse effects on lipid profiles in individuals with PCOS with obesity [77], longer term studies are warranted. Additionally, this study lacked a description of the demographic characteristics and a control group. Furthermore, this type of intervention may not be appropriate amongst all BMI groups.

#### 3.1.3. Dietary Patterns

Low-Glycemic Index (GI) Diet.

Studies (Table 1) have found a low-GI diet to improve insulin sensitivity [78,79,80], even when the intervention is designed to be isocaloric [79,80] or when clinically significant weight loss does not occur [78]. Individuals across BMI ranges were included in two of these studies [79,80]. In one of these studies, when researchers compared an ad libitum low-fat, low-GI diet to a conventional low-fat healthy diet in individuals with PCOS, participants were advised to follow the intervention until 7% of baseline body weight was lost [78]. However, only 59% and 50% of participants in the low-GI group and the conventional healthy diet group were able to meet that goal, respectively, perhaps due to the ad libitum nature of the study design [78]. Furthermore, the attrition rate was high (49%); participants who dropped out had a higher baseline BMI and higher insulin resistance [78], suggesting that those with a higher prevalence of cardiometabolic risk factors may not be responsive to this type of intervention. Despite a large proportion of participants not achieving the desired weight loss, there were still improvements in insulin sensitivity [78]. In other research exploring isocaloric low-GI diet interventions with individuals across BMI ranges, improvements in insulin sensitivity were observed [79,80], indicating that metabolic changes can reduce cardiometabolic risk in the absence of anthropometric changes. Yet these studies suffer from small sample sizes [79,80], and one study did not have a comparison group [79].

Low-Starch, Low-Dairy Diet.

Researchers examined the effects of a low-starch, low-dairy diet for eight weeks in ten individuals with PCOS with overweight and obesity [81] (Table 1). The ad libitum diet excluded all grains, beans, pulses, dairy, and sugar. After eight weeks, body weight, BMI, WC, hip circumference, fat mass, fat mass percentage, and fasting insulin concentrations significantly decreased [81]. Yet this study presents with several limitations, including a small sample size, lack of control group, and no assessment of pre-intervention dietary intake.

Ketogenic Mediterranean Diet.

A ketogenic Mediterranean diet intervention (Table 1) showed initial improvements in cardiometabolic risk profiles, including lipid profiles after 12 weeks among individuals with PCOS with overweight and obesity [82]. Researchers found significant reductions in body weight, BMI, fat mass, WC, lean body mass (LBM), glucose and insulin concentrations, HOMA-IR, and lipid profiles, along with improvements in HDL-C concentrations, among individuals with PCOS after the 12-week intervention. The researchers initially described the intervention as eucaloric but later described it as a low-calorie ketogenic diet, making it unclear how the energy intake was modified. Furthermore, the study lacked a description of the demographic characteristics of participants and control group. Additionally, it is unclear if this is an appropriate dietary pattern for individuals with PCOS at a healthy BMI. Ultimately, the long-term effects and sustainably of this type of dietary pattern are unknown.

#### 3.1.4. Eating Behavior

Meal Timings/Frequency.

Studies (Table 1) have explored the effects of a restricted feeding period (fasting 4 pm–8 am) [83], Ramadan fasting [86], modification of energy intake timing (high calorie breakfast versus high calorie dinner) [84], and a three or six meals per day pattern [85] for individuals with PCOS. All four interventions were designed without energy manipulation [83,84,85,86], with three of the studies including individuals across BMI ranges [84,85,86]. Three of the studies found improvements in cardiometabolic risk factors [83,84,85], with the one quasi-experimental study finding no differences in cardiometabolic risk factors pre- and post-Ramadan fasting [86]. Yet in the restricted feeding period study, where participants fasted from 4 pm to 8 am daily, only 20% of participants reported never feeling hungry [83]. Feelings of hunger need to be considered when advising patients, as this may limit the long-term sustainability of this type of intervention. A longer study with a control group, larger sample, and larger diversity of participants is needed.

In the study that modified caloric intake timing and only included individuals with a healthy BMI, researchers found significant improvements in cardiometabolic risk factors in the high-calorie breakfast group compared to the high-calorie dinner group [84]. The low attrition rate of the study (7% in the breakfast group, 10% in the dinner group) is also a strength and a possible indicator that this intervention is more realistic and sustainable [84]. Furthermore, it provides evidence of an intervention for individuals with a healthy BMI and for whom weight loss interventions described in the literature are not appropriate.

Modification of Advanced Glycation End Products (AGEs).

Researchers explored the effect of advanced glycation end products (AGEs) on metabolic profiles in a study that included individuals with PCOS across BMI ranges (Table 1) [87]. Participants increased their cooking temperatures during the two-month high AGEs phase and decreased their cooking temperatures during the two-month low AGE phase. Serum AGEs were correlated with insulin concentrations and HOMA-IR during the low AGEs diet, indicating that changes in dietary AGE intake were related to changes in insulin sensitivity. Limitations of the study include no washout period and a high dropout rate (32%).

The variety of interventions altering dietary patterns makes it challenging to identify the most effective dietary intervention for individuals with PCOS. Overall, many of the eucaloric interventions have similar improvements in cardiometabolic risk factors compared to hypocaloric interventions, suggesting that changes in dietary patterns without caloric restriction may promote beneficial changes for individuals with PCOS. Furthermore, many of these changes in the dietary patterns would be appropriate for individuals with a range of BMIs.

#### 3.1.5. Energy Restriction and Macronutrient Distribution

Energy Restriction with Protein Modification.

Several studies (Table 1) have looked at restricting energy and modifying protein intake in individuals with PCOS with overweight and obesity. One study included individuals across BMI ranges for whom they modified the estimated energy needs, yet most of the study participants had a BMI greater than or equal to 25 kg/m^2^ [91]. These energy-restricted with protein modification interventions included a comparison of a hypocaloric diet with protein supplement compared to a hypocaloric diet with simple sugar supplement [88], a hypocaloric high versus low-protein diet [89,91], and a hypocaloric high-protein versus high-CHO diet [92].

While most studies found no significant effect of dietary composition on cardiometabolic risk factors [89,91,92], one study found a hypocaloric diet with a powdered protein supplement to decrease body weight, fat mass, and total cholesterol concentrations compared to a hypocaloric diet with a simple sugar supplement [88]. Unfortunately, a decrease in lean body mass [89] and HDL-cholesterol concentrations [88] was reported in two of the interventions. Overall, the results of these interventions need to be interpreted cautiously. All studies had relatively small sample sizes [88,89,91,92], and three lacked a control group [88,89,92] and were short in duration [88,91,92], thus limiting the understanding of the sustainability and long-term effects of these dietary interventions. Furthermore, the lack of participant diversity [89,91] limits the generalizability. Lastly, the studies largely included individuals with overweight and obesity, thus further limiting the generalizability of this intervention to individuals with PCOS across BMI ranges.

Energy Restricted with Carbohydrate/Fat Restriction.

Researchers studied the effect of an energy-restricted diet where two meals per day were replaced with a meal replacement beverage, followed by a 24-week maintenance phase with either a CHO counting (<120 g/CHO/day) or fat counting (<50 g/fat/day) protocol in individuals with PCOS with overweight and obesity [93]. While total cholesterol, triglyceride, LDL-C, and C-reactive protein concentrations significantly decreased after phase one, these variables increased during phase two. Thus, there was no difference in these variables from baseline to study completion [93], perhaps highlighting that the cardiometabolic benefits from weight loss on meal replacements alone are difficult to maintain. Weight regain was reported during phase two, but a significant weight loss was maintained from baseline [93]. The high attrition rate and weight regain [93] question the sustainability and acceptability of these diets. A large limitation of the study [93] is the lack of diversity in the sample as well as the lack of generalizability to individuals across BMI ranges.

#### 3.1.6. Energy Restriction and Dietary Pattern

Energy-Restricted Dietary Approaches to Stop Hypertension (DASH) Diet.

Two studies (Table 1) explored an energy-restricted DASH diet compared with an energy-restricted control diet in PCOS individuals with overweight and obesity [94,95]. Significant improvement in weight, BMI, and insulin concentrations were seen in the energy-restricted DASH diet group in both studies [94,95]. In addition, one of the studies found the energy-restricted DASH diet to significantly decrease triglycerides and very low-density lipoprotein cholesterol concentrations compared to the control group [94]. Thus, the energy-restricted DASH diet demonstrated an ability to improve cardiometabolic risk factor profiles above and beyond energy restriction alone, yet this type of diet may not be suitable for all individuals with PCOS, particularly those within a healthy BMI range.

Energy-Restricted Low-Glycemic Index (GI) Diet.

In addition to the low-GI studies discussed above, studies have examined an energy-restricted, low-GI diet for individuals with PCOS. The low GI interventions included an energy-restricted, low-GI diet [96,97,98] and an energy-restricted, low-GI, high-protein diet [99]. Two of the studies included individuals with PCOS with overweight and obesity [96,99]. One of the studies included individuals with a healthy BMI (21% of participants), yet all were given the recommendation to reduce caloric intake by 600 kcal/day [97,98].

Researchers found that a hypocaloric diet with modification of both GI and protein content has beneficial effects on cardiometabolic risk factors compared to a hypocaloric diet on its own [99]. However, the study modified both the GI and percentage of energy coming from protein, making it unclear which one of those factors, or the combination of both, drove the results. The limitations of these studies, similar to many of the other studies included in this review, include the lack of a control group [97,98], different measures of cardiometabolic risk [96], and the inability to determine what is driving the relationship of the improvement in cardiometabolic risk factors.

Overall, a large variety of dietary interventions have been studied in the research literature. While some of the interventions have limited the energy intake substantially (VLCDs), more modest energy-restricted diets (500 calories/day), as well as changes in dietary patterns without energy restriction (such as, low carbohydrate, high protein, and low glycemic index) and alterations in eating behavior (such as modification of caloric intake timing, time-restricted feeding, and number of meals per day), appear to offer similar benefits compared to greater energy restrictions. Interventions that modified the dietary pattern without energy restriction were found to improve cardiometabolic risk factors in individuals with PCOS across BMI ranges, indicating an approach that might be realistic for most individuals with PCOS. Yet many of the studies suffer from the same limitations including small sample sizes, a short duration of the interventions, a lack of control groups, high attrition rates, and a lack of randomized controlled trials.

### 3.2. Lifestyle Interventions

The research literature has examined lifestyle interventions in the forms of diet (energy-restricted diets, dietary patterns, nutrition education, and energy restriction with dietary patterns) and another behavioral modification (e.g., exercise or cognition) for individuals with PCOS, largely focusing on individuals with overweight and obesity. The findings are summarized in Table 2.

#### 3.2.1. Energy Restriction and Exercise

Researchers have explored lifestyle interventions that included energy-restricted diets and exercise in individuals with PCOS with overweight and obesity (Table 2). The interventions included in this review differ in the amount of energy restricted, with energy deficits ranging from 500–800 kcal/day deficit [100,101,102,103,104] or total energy intakes ranging between 1000–1400 kcal/day [105,106,107]. The exercise components also varied and consisted of individualized exercise programs [100,101,102], structured exercise training programs [103,106], and general exercise recommendations [104,105,107].

Overall, energy restriction plus exercise has been shown to decrease weight [104,105,106], BMI [101,102,104,105], WC [101,102,104,106,107], hip circumference [104], body fat [105], fat-free mass [105], lean body mass [101,102], blood pressure [104,106], HOMA-IR [106,107], glucose [106], insulin [106], triglycerides [104], total cholesterol [101,102,106], and LDL-C concentrations [104,106], and to improve HDL-C concentrations [107] in individuals with PCOS. The reduction in lean body mass, even in the presence of exercise, warrants further study. Additionally, some of these initial changes may be difficult to maintain. After three months of an energy-restricted (600 kcal/day deficit) and physical activity (30 min, two to three times per week) intervention, individuals with PCOS had significantly lower body weight, BMI, WC, hip circumference, WHR, HOMA-IR, LDL-C, and insulin concentrations compared to baseline [104]. Yet at six months, the changes in insulin concentrations and HOMA-IR were no longer significant, while significant decreases in systolic blood pressure and triglyceride concentrations emerged [104]. Further study of the long-term effects of lifestyle interventions is needed.

In two studies [100,103], participants were stratified by ovulation status. Participants who resumed ovulation had decreased body weight [100,103] and abdominal fat [100]. Yet these two studies differed in their intervention, with one being a six-month energy restriction of at least 500 kcal/day with individualized exercise programs [100] and the other comparing a structured exercise training program or a higher protein diet with 800 kcal/day deficit [103]. These results are difficult to compare with other studies that do not stratify their results based on ovulation status. Additionally, in one of these studies [103], participants self-selected into their intervention group, which may have introduced selection bias into the study.

Researchers looking at diet only compared with diet and aerobic exercise or diet and combined aerobic–resistance exercise found that the addition of a structured exercise component did not improve clinical and biochemical cardiometabolic risk preferentially over a diet only intervention but led to significantly greater improvements in anthropometric measures [106]. Thus, having both diet and exercise components may have larger improvements in cardiometabolic risk profiles in individuals with PCOS than either diet or exercise alone. Yet these studies focused on individuals with PCOS with overweight and obesity, making them less generalizable to individuals with PCOS across BMI ranges.

#### 3.2.2. Dietary Patterns and Exercise

One study included in this review (Table 2) looked at changes in dietary patterns and exercise in improving cardiometabolic risk in individuals with PCOS across BMI ranges. Researchers compared a low-glycemic index pulse-based diet, high in beans and legumes, or the therapeutic lifestyle change diet, focused on increased fiber and decreased saturated fat and cholesterol intakes, in individuals with PCOS for one year [108]. Both of these dietary interventions were paired with aerobic training programs. Initial changes to cardiometabolic risk included a decrease in BMI, WC, systolic blood pressure (SBP), glucose, insulin, and total cholesterol concentrations in both the pulse-based and therapeutic lifestyle changes groups. The pulse-based group initially had a significantly larger decrease in DBP, insulin area under the curve, triglyceride, LDL-C, and total cholesterol/HDL-C ratio concentrations compared to the therapeutic lifestyle changes diet. At 12 months, only the increased HDL-C concentrations and lower total cholesterol/HDL-C ratio stayed improved in the pulse-based group. Other improvements in cardiometabolic risk returned to pre-intervention levels [108]. Unfortunately, this study had a high attrition rate and poor response rate to dietary recalls [108]. Additionally, although the study protocol included individuals with PCOS across BMI ranges, the baseline mean BMI was 32.5 ± 8.4 kg/m^2^ and 33.3 ± 9.0 kg/m^2^ for the pulse-based and therapeutic lifestyle changes groups, respectively [108]. Thus, greater understanding of the effect of this type of lifestyle change in individuals with PCOS of all body sizes is needed.

#### 3.2.3. Nutrition Education and Exercise

Studies that combine nutrition education and exercise have been conducted for individuals with PCOS with overweight and obesity (Table 2). The two studies included in this review provided nutrition education through weekly seminars paired with endurance and resistance training [109], or group and individual nutritional counseling paired with exercise counseling in individuals with PCOS among overweight and obese BMI categories [110]. Researchers examining the effects of the weekly seminars paired with exercise found a decrease in insulin concentrations, despite no significant decrease in BMI. This highlights a possible cardiometabolic benefit of a nutrition education and exercise intervention in the absence of weight loss [109].

In a similar study examining individual or group diet and exercise counseling, those participants who resumed ovulation had a decrease in WC, central abdominal fat, and insulin concentrations, with an increase in insulin sensitivity [110]. Researchers did not assess the difference in results between group and individual counseling [110], limiting our understanding of which intervention delivery method may be more effective. Additionally, the participants in these two studies were all categorized as overweight or obese by BMI. Furthermore, the small sample sizes of both of these interventions are limitations [109,110].

#### 3.2.4. Energy Restriction, Dietary Pattern, and Exercise

Energy-Restricted Mediterranean-Style Diet, Low-Glycemic Load, and Exercise.

Using a quasi-experimental design, researchers explored a Mediterranean-style, low-glycemic load, hypocaloric diet with physical activity recommendations for 12 weeks in individuals with PCOS and overweight and obesity [111]. The dietary pattern comprised 25% protein, 25% fat, and 50% CHO and was designed to be low-fat, with moderate-to-high fiber and an energy deficit of 500 kcal/d. Researchers found decreased BMI, WC, body fat percentage, systolic blood pressure, diastolic blood pressure, HOMA-IR, glucose, insulin, total cholesterol, LDL-C, triglycerides, C-reactive protein concentrations, and triglycerides/HDL-C ratio [111]. Yet there was no comparison group, compliance with the physical activity regimen was not assessed, and researchers only included participants with overweight or obesity. Thus, the appropriateness of this lifestyle intervention for individuals with PCOS across all BMI categories cannot be determined.

Energy-Restricted High Protein, Low Carbohydrate Diet and Exercise.

Researchers assessed a 3-month energy-restricted, high-protein, low-carbohydrate diet and aerobic physical activity in individuals with PCOS with overweight and obesity [112]. The intervention consisted of an unspecified amount of energy restriction for weight loss in addition to following a high-protein, low-carbohydrate (40% CHO, 30%, 30% protein) dietary pattern with 45 min of aerobic activity two to three times/week [112]. Post-intervention body weight, BMI, and insulin concentrations significantly decreased [112], highlighting potential cardiometabolic benefits of this intervention. Yet similar to the dietary interventions that combined dietary patterns and energy restriction, it is unclear if one aspect is driving the results. Furthermore, the energy restriction implemented in this lifestyle intervention would not be suitable for individuals with PCOS with a healthy body weight.

#### 3.2.5. Energy Restriction and Cognition

Two studies included in this review examined energy restriction combined with a cognitive behavior therapy (CBT) intervention in individuals with PCOS with overweight and obesity (Table 2). In one study, when researchers incorporated CBT as an adjunct to lifestyle counseling, they found significant improvements in weight loss compared to standard lifestyle counseling alone [113]. Additionally, participants in the CBT group were significantly more likely to meet their exercise goal and keep their weekly food diary than those solely in the lifestyle modification group [113]. These results highlight the potential of psychological interventions in improving cardiometabolic outcomes. Yet this study had a high attrition rate for the CBT group, a small sample size, only included participants with an overweight or obese BMI, and was of the shortest duration (8 weeks) of the lifestyle interventions included in this review.

A retrospective analysis of a lifestyle intervention of individuals with PCOS with obesity incorporated a VLCD of approximately 600 calories per day and a behavior change program informed by CBT [114]. After 12 weeks, the PCOS group had lower body weight compared to baseline [114]. This study was limited in that it was a retrospective analysis of a lifestyle intervention, had a high attrition rate (73%), did not compare dietary intake data, and only included participants with PCOS with obesity [114].

**Table 2:** Lifestyle intervention studies to improve cardiometabolic risk in individuals with polycystic ovary syndrome.

Reference	Design	Participants	Intervention	Cardiometabolic Outcomes	Results/Conclusions	Covariates Considered	Limitations
Energy Restriction and Exercise Energy-Restricted Diets and Exercise							
Kuchenbecker and colleagues (2011) [100]	Pilot study	A total of 32 anovulatory individuals with PCOS ^a^ (BMI ^b^ > 29.0 kg/m^2^), <38 years, diagnosed by Rotterdam criteria.	Lifestyle program (6 months): Individualized dietary advice (500 kcal/d energy deficit); Individualized exercise program.	Anthropometric measures and insulin; ovulation status (resumed ovulation or did not resume ovulation).	Compared to anovulation group, ovulation group showed the following: ↓ body weight (resumed ovulation: −6.3%, did not resume ovulation: −3.0%; *p* = 0.018) and abdominal fat (resumed ovulation: −15.0%, did not resume ovulation: −4.3%; *p* = 0.025).	Stratified by ovulation status.	High attrition rate (25%), no comparison group, and lack of diverse sample.
Nybacka and colleagues (2017) [101,102]	Randomized three-arm parallel study	A total of 57 individuals with PCOS (BMI > 27.0 kg/m^2^), age 18–40 years, diagnosed by Rotterdam criteria.	Lifestyle program (4 months): Energy-restricted diet (at least 600 kcal/d energy deficit); or Individually adjusted exercise program; or Energy restriction plus exercise.	Anthropometric measures, glucose, insulin, lipid profile, and CRP ^c^.	Compared to the other two groups, the energy restricted group showed the following: ↓ body fat (pre: 45.5 ± 3.6 kg, post: 44.0 ± 6.0 kg; *p* < 0.05), HOMA-IR ^d^ (pre: 3.6(3.0–6.9), post: 2.9(2.2–3.6); *p* < 0.05), and LDL-C ^e^ (pre: 2.8 ± 0.8 mmol/L, post: 2.3 ± 0.9 mmol/L; *p* < 0.05). Compared to the other two groups, the exercise group showed the following: ↓ CRP (pre: 5.1 ± 6.8 mg/L, post: 3.8 ± 3.5 mg/L; *p* < 0.05). Compared to the exercise group, both energy restricted and energy restricted plus exercise groups showed the following: ↓ BMI (energy restricted: −6%, *p* < 0.001; energy restricted plus exercise: −5%, *p* < 0.001), WC ^f^ (energy restricted: pre: 103.8 ± 13.0 cm, post: 95.5 ± 13.2 cm, *p* < 0.001; energy restricted plus exercise: pre: 110.3 ± 14.6 cm, post: 106.6 ± 14.6 cm, *p* < 0.05) LBM ^g^ (energy restricted: pre: 50.1 ± 7.7 kg, post: 46.7 ± 5.3 kg, *p* < 0.05; energy restricted plus exercise: pre: 52.9 ± 8.4 kg, post: 49.7 ± 7.6 kg, *p* < 0.001), and TC ^h^ (energy restricted: pre: 4.5 ± 1.0 mmol/L, post: 4.0 ± 1.4 mmol/L, *p* < 0.01; energy restricted plus exercise: pre: 4.7 ± 1.1 mmol/L, post: 4.3 ± 1.1 mmol/L, *p* < 0.05).	Age, BMI, and body composition.	
Olszanecka-Glinianowicz and colleagues (2008) [105]	Non-randomized study	A total of 15 individuals with PCOS (BMI > 30 kg/m^2^), diagnosed by hormonal and ultrasonographic diagnosis of PCOS.	Lifestyle program (unknown duration): Hypocaloric diet (1000–1200 kcal) with limited intake of simple CHO ^i^ and animal fats; Regular exercise (30 min 3×/week).	Anthropometric measures, glucose, insulin, and lipid profile.	Compared to baseline: ↓ body weight (pre: 96.6 ± 17.0 kg, post: 84.7 ± 14.5; *p* < 0.00001), BMI (pre: 36.1 ± 6.6 kg/m^2^, post: 31.6 ± 5.8 kg/m^2^; *p* < 0.00001), body fat (pre: 42.6 ± 12.5 kg, post: 35.5 ± 11.2 kg; *p* < 0.00001), and LBM (pre: 53.8 ± 8.1 kg, post: 49.1 ± 4.7 kg; *p* < 0.01); ↓ TG ^j^ (pre: 1.4 ± 0.7 mmol/L, post: 1.1 ± 0.4 mmol/L; *p* < 0.05); No sig. difference between insulin, glucose, HOMA-IR, or other lipids.		Does not indicate how long the intervention lasted, no comparison group, small sample size, and does not specify PCOS diagnostic criteria used.
Palomba and colleagues (2008) [103]	Non-randomized pilot study	A total of 40 anovulatory infertile individuals with PCOS (BMI 30.0–35.0 kg/m^2^), ages 18–35 years, diagnosed by Rotterdam criteria.	Lifestyle program (24 weeks): Structured exercise training program (three training sessions per week); or Energy-restricted high-protein diet (35% protein, 45% CHO and 20% fat; with 800 kcal deficit per day).	Anthropometric measures and glucose, insulin; ovulation status (resumed ovulation or did not).	Compared to the ovulatory structured exercise training group, the ovulatory energy-restricted, high-protein diet group showed the following: ↓ body weight (−10.5 ± 4.1 kg; *p* < 0.05) and BMI (−15.4 ± 3.92 kg/m^2^; *p* < 0.05). Compared to ovulatory energy-restricted high-protein diet group, the ovulatory structured exercise training group showed the following: ↓ WC (−9.6 ± 2.1 cm; *p* < 0.05), insulin (−23.4 ± 10.0 pmol/L; *p* < 0.05), and HOMA-IR (−41.0 ± 15.9; *p* < 0.05).	Stratified by ovulation status.	Participants self-selected intervention and the study did not include lipid profiles.
Pasquali and colleagues (2011) [107]	Retrospective analysis of lifestyle intervention	A total of 65 individuals with PCOS, (BMI > 25 kg/m^2^), diagnosed by 1990 National Institute of Health criteria.	Lifestyle program: Hypocaloric diet (1200–1400 kcal/d) (6 months); Followed by a mildly restricted intake (500 kcal/d deficit) plus a daily walk for 30 min 5×/week (10–67 months).	Anthropometric measures, glucose, insulin, and lipid profile; recovery of PCOS based on diagnostic criteria (persisting PCOS; partial recovery; full recovery).	Compared to baseline: ↓ WC (persisting PCOS: −14.1 ± 12.2 cm, partial recovery: −9.3 ± 11.5 cm, fully recovered −9.9 ± 6.0 cm; *p* < 0.001) and HOMA-IR (persisting PCOS: −1.8 ± 2.6, partial recovery: −1.8 ± 3.0, fully recovered −1.3 ± 1.2; *p* < 0.05); ↑ HDL-C ^k^ (persisting PCOS: 9.2 ± 6.9 mg/dL, partial recovery 4.8 ± 9.9 mg/dL, fully recovered: 3.5 ± 11.2 mg/dL; *p* < 0.05). Compared to the persisting PCOS group, both partial recovery and full recovery groups showed the following: ↓ TC (persisting PCOS: −5.8 ± 20.2 mg/dL, partial recovery: −14.6 ± 41.2 mg/dL, full recovery: −15.4 ± 35.1 mg/dL; *p* < 0.01) and glucose (persisting PCOS: −4.6 ± 7.9 mg/dL, partial recovery: −5.5 ± 12.0 mg/dL, full recovery: −6.0 ± 11.1 mg/dL; *p* < 0.01).	Stratified by recovery of PCOS.	No control group, did not measure LDL cholesterol, did not include those who did not lose body weight or did not comply with intervention, and no comparison of dietary intake.
Thomson and colleagues (2008) [106]	Randomized parallel study	A total of 94 sedentary individuals with PCOS, (BMI: 25.0–55.0 kg/m^2^), age 18–41 years, diagnosed by Rotterdam criteria.	Lifestyle program (20 weeks): Energy-restricted, high-protein diet (5000–6000 kJ/d [1195–1434 kcal/d]; 30% protein, 40% CHO, 30% fat); or Energy-restricted, high-protein diet and aerobic exercise (walking/jogging program 5×/week); or Energy-restricted, high-protein diet and combined aerobic–resistance exercise (progressive resistance training 2×/week).	Anthropometric measures, insulin, glucose, and lipid profile.	Compared to baseline, all groups showed the following: ↓ body weight (−9.4 ± 1.9 kg), WC (−11.1%), BP ^l^ (−5.6 ± 2.7 mmHg), glucose (−0.2 mmol/liter), insulin (−4.3 mIU/L), TC (−0.5 mmol/L), TG (−0.4 mmol/L), and LDL-C (−0.1 mmol/L), with no sig. differences between groups. Compared with diet only group, both diet and aerobic exercise and diet and combined exercise group showed the following: ↓ BF% ^m^ (diet and aerobic: −4.4 ± 2.8%, diet and combined: −3.2 ± 2.9%; *p* < 0.03), fat mass (diet and aerobic: −7.5 ± 3.9 kg, diet and combined: −6.7 ± 4.0 kg; *p* < 0.03), and FFM ^n^ (diet and aerobic: −1.2 ± 3.3 kg, diet and combined: −1.8 ± 3.4 kg; *p* < 0.03).		
Wang and colleagues (2021) [104]	Post-intervention follow-up of randomized controlled trial	A total of 87 infertile individuals with PCOS and 172 infertile controls (BMI ≥ 29.0 kg/m^2^), 18–39 years; PCOS diagnosed by Rotterdam criteria.	Lifestyle program (6 months): Energy restriction (600–1200 kcal/d); Physical activity (30 min, 2–3×/week).	Anthropometric measures, blood pressure, glucose, insulin, lipid profile, and CRP.	At 3 months, both groups showed the following: ↓ body weight (−0.5 kg), BMI (−0.3 kg/m^2^), SBP ^o^ (−0.9 mmHg), TG (−0.0 mmol/L), TC (−0.0 mmol/L), HDL-C (−0.0 mmol/L), LDL-C (−0.1 mmol/L), CRP (−0.2 mg/L), and insulin (−16 pmol/L), with no sig. difference between groups. At 6 months, both groups showed the following: ↓ WHR ^p^ (−0.02), SBP (−7.2 mmHg), TG (−0.1 mmol/L), TC (−0.1 mmol/L), HDL-C (−0.03 mmol/L), LDL-C (−0.1 mmol/L), and CRP (−2.3 mg/L), with no sig. difference between groups. Compared to control group, at 6 months, PCOS group showed the following: ↓ DBP ^q^ (−5.2 mmHG, 95% CI: −10.3 to −0.1; *p* = 0.04).	Age.	Non-randomization in the post hoc analysis.
Dietary Pattern and Exercise Pulse-Based Diet with Aerobic Training Program							
Kazemi and colleagues (2018) [108]	Single-blind parallel group randomized controlled trial	A total of 95 individuals with PCOS, ages 18–35 years, diagnosed by irregular periods, unwanted male-pattern facial and/or body hair growth, and infertility.	Lifestyle program (16 weeks, follow-up of 1 year): Low-glycemic index, pulse-based diet (high in beans and legumes) with aerobic training program; or TLC ^r^ diet (focused on increased fiber and decreased saturated fat and dietary cholesterol intake) with aerobic training program.	Anthropometric measures, lipid profile, CRP, HbA1c ^s^, glucose, and insulin.	Compared to baseline, at 16 weeks, both groups showed the following: ↓ BMI (pulse based: −1.3 ± 1.4 kg/m^2^, TLC: −1.8 ± 6.1), WC (pulse based: −4.4 ± 11.2 cm, TLC: −1.7 ± 7.6 cm), trunk fat mass (pulse based: −1.1 ± 2.0 kg, TLC: −2 ± 3.9), body fat (pulse based: −1.7 ± 2.4 kg, TLC: −3.0 ± 7.5 kg), BF% (pulse based: −1.0 ± 2.0%, TLC: −1.0 ± 2.4%), SBP (pulse based: −3 ± 8 mmHg, TLC: −5 ± 8 mmHg), glucose (pulse based: −0.4 ± 1.7 mmol/L, TLC: −0.8 ± 1.5 mmol/L), insulin (pulse based: −4.0 ± 9.7 μIU/mL, TLC: −3.0 ± 6.8 uIU/mL), HOMA-IR (pulse based: −1.0 ± 2.1, TLC: −1.3 ± 2.1), and TC (pulse based −0.4 ± 0.5 mmol/L, TLC: −0.1 ± 0.5 mmol/L), with no sig. difference between groups. Compared to TLC group, the pulse-based group showed the following: ↓ DBP (−3.6 ± 6.7 mmHg; *p* = 0.05), AUCins ^s^ (−121.0 ± 229.9 μIU/mL × min; *p* = 0.05), TG (−0.2 ± 0.6 mmol/L; *p* = 0.04), LDL-C (−0.2 ± 0.4 mmol/L; *p* = 0.05), and TC/HDL-C ratio (−0.4 ± 0.4; *p* < 0.001); ↑ HDL-C (0.1 ± 0.2 mmol/L; *p* < 0.01). Compared to the TLC group, at 6 months, the pulse-based group showed the following: ↓ TC/HDL-C ratio was maintained (*p* = 0.02). Compared to 16 weeks, at 6 months: ↑ body weight (pulse based—16 weeks: 78.0 ± 12.6 kg, 6 months: 79.6 ± 13.4 kg; TLC—16 weeks: 92.0 ± 20.5 kg, 6 months: 94.8 ± 18.1 kg), BMI (pulse based:—weeks: 29.0 ± 5.6 kg/m^2^, 6 months: 31.2 ± 7.2 kg/m^2^; TLC—16 weeks: 33.9 ± 8.9 kg/m^2^, 6 month: 34.7 ± 8.8 kg/m^2^), insulin (pulse based—16 weeks: 9.8 ± 8.7 μIU/mL, 6 months:13.3 ± 11.2 μIU/mL; TLC—16 weeks: 10.4 ± 11.9 μIU/mL, 6 months: 16.7 ± 9.1 μIU/mL), and TC (pulse based—16 weeks: 4.4 ± 0.8 mmol/L, 6 months: 4.6 ± 0.8 mmol/L; TLC—16 weeks: 4.2 ± 0.8 mmol/L, 6 months: 4.4 ± 0.7 mmol/L), with no sig. differences between groups. Compared to TLC group, at 12 months, the pulse-based group showed the following: ↑ HDL-C (*p* = 0.02). ↓ TC/HDL-C ratio was maintained (*p* = 0.04).	Metformin (stratified and randomized separately).	High attrition rate (33% in each group), no control group, poor response rate to dietary recalls, and did not follow set PCOS diagnostic criteria.
Nutrition Education and Exercise Nutrition Education or Counseling and Exercise							
Bruner and colleagues (2006) [109]	Randomized controlled trial	A total of 12 sedentary individuals with PCOS (BMI > 27.0 kg/m^2^), diagnosed by Rotterdam criteria,	Lifestyle program (12 weeks): Nutrition counseling (weekly nutritional seminars); or Nutrition counseling and exercise (endurance and resistance training).	Anthropometric measures, insulin, and lipid profile.	Compared to baseline, at 12 weeks: ↓ WC (nutrition counseling: −5%, nutrition counseling and exercise: −5.3%) and insulin (nutrition counseling: −55%, nutrition and exercise counseling: −29%), with no sig. differences between groups.		Small sample size.
Huber-Buchholz and colleagues (1999) [110]	Prospective study	A total of 18 anovulatory individuals with PCOS and normal glucose tolerance and 10 controls, (BMI 27.0–45.0 kg/m^2^); PCOS diagnosed by Rotterdam criteria.	Lifestyle program (6 months): Group dietary and exercise counseling and support; or Individual dietary and exercise counseling and support.	Anthropometric measures, glucose tolerance, and insulin; ovulation (responder) or no ovulation (non-responder).	Compared to the anovulatory group, the ovulation group showed the following: ↓ WC (responders: 102 ± 3.0 cm^2^, non-responders: 106 ± 4.3; *p* = 0.03), central abdominal fat (*p* = 0.04), and insulin (responders: 13.6 ± 1.7 nmol/L, non-responder: 23.0 ± 3.5 nmol/L; *p* = 0.003); ↑ insulin sensitivity (71%; *p* < 0.05).	Stratified by ovulation.	Small sample size; did not assess difference between group and individual intervention.
Energy Restriction, Dietary Pattern, and Exercise Energy-Restricted Mediterranean-Style Anti-Inflammatory Diet, Low-Glycemic Load, and Exercise							
Salama and colleagues (2015) [111]	Quasi-experimental trial	A total of 100 individuals with PCOS, (BMI 25–40 kg/m^2^), ages 20–40 years, diagnosed by Rotterdam criteria.	Lifestyle program (12 weeks): Mediterranean-style anti-inflammatory, low-glycemic load, hypocaloric diet (500 kcal/d deficit); Physical activity recommendations (stairs and sit-ups).	Anthropometric measures, blood pressure, glucose, insulin, lipid profile, and CRP.	Compared to baseline: ↓ BMI (−7.1%; *p* < 0.001), WC (−6.6%; *p* < 0.001), BF% (−9.2%; *p* < 0.001), SBP (pre: 124 ± 9 mmHg, post: 121 ± 6 mmHg; *p* < 0.001), DBP (pre: 82 ± 6 mmHg, post: 80 ± 5; *p* < 0.001), glucose (pre: 92 ± 10 mg/dL, post: 86 ± 9; *p* < 0.001), insulin (pre: 14.8 ± 8.5 μIU/mL, post: 9.7 ± 5.3 μIU/mL; *p* < 0.001), HOMA-IR (−27.5%; *p* < 0.001), TC (pre: 199 ± 39 mg/dL, post: 178 ± 29 mg/dL; *p* < 0.001), LDL-C (130 ± 36 mg/dL, post: 113 ± 28 mg/dL; *p* < 0.001), TG (pre: 105 ± 37 mg/dL, post: 79 ± 27; *p* < 0.001), TG/HDL-C ratio (pre: 2.4 ± 1.2, post: 1.7 ± 0.8; *p* < 0.001), and CRP (pre: 9.6 ± 9.4 mg/L, post: 5.1 ± 3.8; *p* < 0.001).		No comparison group; did not measure compliance with physical activity regimen.
Energy-Restricted, High-Protein, Low-Carbohydrate Diet and Exercise							
Ujvari and colleagues (2014) [112]	Non-randomized experiment	A total 18 individuals with PCOS (BMI > 27.0 kg/m^2^), ages 18–40 years, diagnosed by Rotterdam criteria.	Lifestyle program (3 months): Energy-restricted, high-protein, low-CHO (40% CHO, 30% fat, 30% protein) diet; Physical activity (aerobic activity for 45 min 2–3×/week).	Anthropometric measures, blood pressure, glucose, insulin, and QUICKI ^t^.	Compared to baseline: ↓ body weight (pre: 103.8 ± 15.0 kg, post: 98.7 ± 15.9 kg; *p* < 0.001), BMI (pre: 37.0 ± 5.7 kg/m^2^, post: 35.2 ± 5.6 kg/m^2^; *p* < 0.001), insulin (pre: 42.6 (27.4–96.4) mIU/L, post: 30.9 (21.3–59.1) mIU/L; *p* < 0.01), and QUICKI (pre: 0.27 ± 0.03, post: 0.29 ± 0.03; *p* < 0.05).		Small sample; no comparison group for the intervention.
Energy-Restricted Diet and Psychological Intervention Energy-Restricted Diet and Cognitive Behavior Therapy							
Cooney and colleagues (2018) [113]	Randomized clinical pilot trial	A total of 33 individuals with PCOS (BMI 27.0–50.0 kg/m^2^) and a positive screen for depressive symptoms; PCOS diagnosed by 1990 National Institute of Health criteria.	Lifestyle program: Weekly nutrition/exercise counseling (16 weeks); or Weekly nutrition/exercise counseling plus CBT ^u^ (8 weeks).	Anthropometric measures, lipid profile, glucose, insulin, and CRP.	Compared to nutrition/exercise counseling alone, weekly nutrition/exercise counseling plus CBT showed the following: ↓ body weight (weekly counseling plus CBT: −0.35 kg/week [95% CI 0.47 to 0.23], weekly counseling: −0.16 kg/week [95% CI: 0.28 to 0.04]; *p* = 0.033); More likely to meet their exercise goal (weekly counseling plus CBT: 59%, weekly counseling: 38%; *p* = 0.002) and to keep weekly food diary (weekly counseling plus CBT: 83%, weekly counseling: 66%; *p* = 0.007).Compared to nutrition/exercise counseling plus CBT, nutrition/exercise counseling alone showed the following: ↓ TC (weekly counseling plus CBT: 3 (−5 to 7) mg/dL, weekly counseling: −19 (−19 to −11) mg/dL; *p* = 0.03).		Small sample size, high dropout rate for CBT group (40%), and short duration.
Nikokavoura and colleagues (2015) [114]	Retrospective analysis of lifestyle intervention	A total of 508 individuals with PCOS and 508 controls, (BMI ≥ 30.0 kg/m^2^), ages 18–75 years.	Lifestyle program (12 weeks): Lighterlife Total intervention (VLCD ^v^ approximately 600 kcal/d); Behavior change program informed by CBT.	Body weight; BP.	Compared to baseline, at 12 weeks: ↓ body weight (PCOS: −18.5 ± 6.6 kg, control: −19.4 ± 5.7 kg) and BMI (PCOS: 40.0 ± 6.4 kg/m^2^ vs. 33.2 ± 6.0 kg/m^2^, control: 40.0 ± 6.3 kg/m^2^ vs. 32.9 ± 5.7 kg/m^2^), with no sig. differences between groups. Compared to control group, the PCOS group showed the following: ↓ SBP (PCOS: −5.5 ± 6.1 mmHg, control: −0.9 ± 6.1 mmHg; *p* < 0.001).	Age and BMI-matched controls.	Retrospective, no information on diagnostic criteria, no information on medication, high attrition rate in the intervention (73%), and no comparison of dietary intake.

↑: Increased; ↓: Decreased; ^a^ PCOS: Polycystic Ovary Syndrome; ^b^ BMI: Body Mass Index; ^c^ CRP: C-Reactive Protein; ^d^ HOMA-IR: Homeostatic Model Asessment of Insulin Resistance; ^e^ LDL-C: Low-Density Lipoprotein Cholesterol; ^f^ WC: Waist Circumference; ^g^ LBM: Lean Body Mass; ^h^ TC: Total Cholesterol; ^i^ CHO: Carbohydrate; ^j^ TG: Triglycerideas; ^k^ HDL-C: High-Density Lipoprotein Cholesterol; ^l^ BP: Blood Pressure; ^m^ BF%: Body Fat Percentage; ^n^ FFM: Free-Fat Mass; ^o^ SBP: Systolic Blood Pressure; ^p^ WHR: Waist-to-Hip Ratio; ^q^ DBP: Diastolic Blood Pressure; ^r^ TLC: Therapeutic Lifestyle Changes; ^s^ AUCins: Area Under the Curve Insulin; ^t^ QUICKI: Quantitative Insulin-Sensitivity Check Index; ^u^ CBT: Cognitive Behavior Therapy; ^v^ VLCD: Very Low-Calorie Diet.

Overall, considering the lifestyle interventions included in this review, the combination of dietary changes with exercise appears to confer an additional cardiometabolic benefit than just one component alone [101,102,103,109], and at times, even in the absence of changes in weight or anthropometric measures [103,109]. The lifestyle intervention research indicates that incorporating both diet and exercise into a well-rounded intervention may lead to greater improvements in cardiometabolic risk profiles.

Yet limitations of the lifestyle interventions should be considered. Many studies lacked a comparison group [100,105,110,111,112], had a small sample size [105,109,110,112,113], suffered from high attrition rates [100,108,113], and did not report on the length of duration [105]. Furthermore, other studies had participants self-select the intervention group [103], introducing selection bias and affecting internal validity. Furthermore, adherence to the lifestyle intervention was not always measured [111]. The external validity of the studies is limited due to the lack of diversity in participants [100], and many of the studies were limited to individuals who were classified as overweight or obese. Future lifestyle intervention research that looks at both the psychological aspects of behavior change, as well as the long-term maintenance of cardiometabolic risk improvements for individuals across BMI ranges, is needed.

## 4. Discussion

### 4.1. Summary

Overall, a variety of lifestyle interventions can be utilized to reduce the prevalence of cardiometabolic risk factors in individuals with PCOS. In this review, randomized control trials were limited: only 13 of the dietary and 6 of the lifestyle interventions were randomized interventions. Additionally, many of the intervention studies lacked control groups, and most studies focused on weight loss and excluded individuals classified within a healthy BMI range. Only 11 of the dietary and 1 of the lifestyle interventions included participants with a healthy BMI in their inclusion criteria. Furthermore, only 16 of the dietary and 3 of the lifestyle interventions did not involve energy restriction. Yet studies that modified dietary patterns without a caloric deficit [72,73,74,75,76,77,78,79,80,81,82,83,84,85,87,108] saw improvements in cardiometabolic risk factors. Thus, less restrictive dietary approaches may be better suited for this population, as they can be applied irrespective of weight and disordered eating history. This finding is supported by the recommendations from the 2023 International Evidenced-Based Guidelines for the Assessment and Management of PCOS that indicate there are benefits to a healthy lifestyle even in the absence of weight loss [1].

Furthermore, the sustainability of weight loss interventions has been questioned [55,56,57], and weight cycling has been identified as an independent risk factor for cardiovascular disease [58,59]. Several weight loss interventions included in this review had high attrition rates [63,64,65,89,92,93,100], signaling that the interventions may not be sustainable in real-world scenarios. This finding, compounded with the disordered eating and poor body image in the PCOS population [50,51,52,53,54], highlights the importance of a more sustainable lifestyle intervention that is not solely focused on reducing energy intake for individuals with PCOS. Due to the possible difficulty to maintain weight loss long term, focusing on dietary patterns is a strategy to improve health outcomes that may be more sustainable and better meet the psychological needs of this population. Dietary patterns, such as low carbohydrate, high protein, low glycemic index, as well as alterations in the eating behavior including time-restricted feeding, modification of caloric intake timing, and the number of meals per day, should be further explored in this population among individuals across BMI ranges.

Additionally, most studies rely on BMI to identify participants, despite a higher prevalence of cardiometabolic risk factors among all individuals with PCOS, regardless of BMI [19,26]. This largely limits our understanding of the best lifestyle practices for individuals with PCOS across body sizes. Expanding our research recruitment outside of medical centers, where individuals with worse clinical presentations may be prevalent [4,18,39], should be prioritized. Moreover, a more robust screening should be conducted to determine metabolic risk, as a singular measure of body size is a flawed diagnostic criterion of one’s overall metabolic health. Individuals who are classified into overweight or obese BMI categories may be metabolically healthy, just as much as individuals classified into a healthy BMI category could be metabolically unhealthy [115]. In support of this, the 2023 International Evidenced-Based Guidelines discuss weight stigma in PCOS. The guidelines acknowledge that while a higher weight and BMI is a risk factor, it is only one indicator of health and other factors should be considered. Furthermore, the guidelines recommend offering options for weight-inclusive care that is focused on promoting lifestyle change without focusing on intentional weight loss [1].

### 4.2. Strengths and Limitations

To our knowledge, this review is the first to summarize the dietary and lifestyle strategies to reduce cardiometabolic risk in individuals with PCOS across body sizes. Additional, the strengths include the inclusion and discussion of a wide variety of dietary and lifestyle interventions to mitigate cardiometabolic risk in individuals with PCOS. Furthermore, this review included studies that examined lifestyle approaches for individuals with PCOS across BMI ranges, increasing the applicability of the results to individuals with PCOS across body sizes. Lastly, a comprehensive search strategy was used and strict exclusion criteria were followed in an attempt to minimize selection bias. The limitations of this review include that only two databases were searched and it did not include conference proceedings, abstracts, government publications, or theses. The studies incorporated in this review also utilized different diagnostic criteria for PCOS. The current recommendations from the 2023 International PCOS guidelines are to use the International Guideline diagnostic criteria, which builds upon the Rotterdam criteria [1]. Finally, the results of these studies were not systematically reviewed and analyzed.

### 4.3. Implications for the Practice

Currently, few of the included interventions have explored the condition through a more holistic, biopsychosocial lens. Overlooking the psychological and social factors may make interventions less sustainable and ultimately, not appropriate for this population. Individuals with PCOS report being largely unsatisfied with the lifestyle recommendations that they receive [116,117,118,119] and believe that that the psychological aspect of PCOS is largely ignored [118]. Recent American Heart Association recommendations highlight the clear association between psychological well-being and cardiovascular disease, demonstrating how improvements in psychological well-being can lead to improvements in cardiovascular health. Conversely, detriments to psychological well-being can negatively affect cardiovascular health [120]. In support of this, recent work in women with overweight and infertility suggests that childhood adversity is associated with poorer health behaviors [121].

There is likely no one-size-fits-all approach to managing PCOS. Thus, a thorough understanding of an individual’s cardiometabolic risk profile, their weight and disordered eating history, level of central adiposity, dietary preferences, lifestyle behaviors, previous experiences with weight loss attempts [122], and potential harm of weight cycling should all be considered when approaching the care of individuals with PCOS. Attention should be given to the psychological components of weight stigma, living in a larger body, and PCOS.

While there does not appear to be one type of lifestyle that is preferable for PCOS, overall, clinicians should promote the adoption of healthy lifestyle behaviors that include adequate physical activity and a varied, flexible diet that meets nutritional needs, individual preferences, and cultural considerations, while avoiding unduly restrictive, unbalanced diet patterns [1]. Overall, a better understanding of what individuals with PCOS want out of their care and the tailoring of their care to meet their individual preferences and goals is needed.

### 4.4. Implications for the Research

While a great deal of research has been conducted on this topic, it is of variable quality and with a great variety in the type of interventions, limiting our ability to come to strong conclusions. Future research could utilize the data more efficiently through prospectively planned pooled analyses. This collaborative approach would allow researchers to combine efforts to elucidate important areas where information is missing. The most recent International Evidence-Based Guidelines may be a good starting point to identify pertinent research questions.

Additionally, it is important to improve the detection of PCOS in the general population. If detection continues to be limited to risk groups with overweight or obesity and/or infertility, individuals with PCOS with a normal body weight may be missed. In an effort to improve the diagnosis of PCOS, the 2023 International Evidence-Based Guidelines has recommended that serum anti-Mullerian hormone (AMH) could be used in lieu of ultrasound for defining PCOS, in conjunction with the pre-existing diagnostic algorithm [1]. Overall, improving detection may allow for an overall better understanding of the spectrum of disease which can guide future intervention strategies. In addition, efforts should be made to include study participants across body sizes to understand their burden of disease, as well as appropriate lifestyle strategies to mitigate cardiometabolic risk.

Lastly, the role of genetics and epigenetics in PCOS needs to be further explored, as genetic variation appears to play an important role in the pathogenesis of PCOS [123]. Animal studies have shown that epigenetic changes in utero can perpetuate PCOS phenotypes across multiple generations [123]. Understanding the role of genetics and epigenetics can aid in elucidating the etiologies of PCOS, the development of targeted therapies, and the transition toward precision medicine for PCOS.

Overall, this review highlights the critical need for a more sustainable lifestyle intervention that meets the needs of individuals with PCOS of varying body weights. Future research is needed that focuses on improving cardiometabolic risk factors through lifestyle approaches that simultaneously incorporate the biological, psychological, and social considerations of this unique population to identify an approach that is realistic, sustainable, and enjoyable.

## Figures and Tables

**Table 1:** Dietary intervention studies to improve cardiometabolic risk in individuals with polycystic ovary syndrome.

Reference	Design	Participants	Intervention	Cardiometabolic Outcomes	Results/Conclusions	Covariates Considered	Limitations
Energy-Restricted Diets Very Low-Calorie Diets							
Kiddy and colleagues (1989) [60]	Intervention	Five individuals with PCOS ^a^ (BMI ^b^ > 30.0 kg/m^2^), diagnosed by presence of hirsutism, polycystic ovaries on ultrasound, and raised serum testosterone or luteinizing hormone (LH), or both, and six controls (mean BMI: 25.5 ± 2.2).	PCOS group: 330 kcal/d for 4 weeks. Control group: 330 kcal/d for 2 weeks.	Anthropometric measures and insulin.	At 2 weeks, both PCOS and control groups: ↓ body weight (−5.2 ± 2.4 kg in PCOS group; −4.3 ± 1.0 kg in control). At 4 weeks, PCOS group ↓ body weight (−7.1 ± 2.8 kg). Compared to the control group, PCOS: ↓ insulin (*p* < 0.05).		Small sample size and short study duration.
Hamilton-Fairley (1993) [61]	Single-arm intervention	Six individuals with PCOS (BMI > 30.0 kg/m^2^), diagnosed by presence of hirsutism, polycystic ovaries on ultrasound, and raised serum testosterone or LH or both.	350 kcal/d for 1 month.	Body weight, glucose, and insulin.	Compared to baseline: ↓ body weight (mean −6.6%, range 2.2–9.2%); ↓ sum of insulin concentration during OGTT ^c^ (pre: 416 ± 34, post: 201 ± 129 mU/L; *p* < 0.03).		Small sample size, short duration, no control group, and no demographic information of participants.
Van Dam and colleagues (2004) [62]	Intervention	A total of 15 individuals with PCOS (BMI > 30.0 kg/m^2^), 20–38 years, diagnosis based on presence of infertility with elevated serum testosterone.	470 kcal/d for ~29 weeks (until participants lost 10% of baseline body weight).	Anthropometric measures, glucose, and insulin; ovulation (responder) or no ovulation (non-responder).	Compared to baseline: ↓ BMI, glucose, insulin, and HOMA-IR ^d^ with no sig. difference between responders and non-responders.	Stratified by responders and non-responders.	Small sample size and no control group.
Kiddy and colleagues (1992) [63]	Intervention	A total of 24 individuals with PCOS (BMI > 25.0 kg/m^2^), diagnosed by polycystic ovaries on ultrasound and raised serum LH or testosterone, or both.	Interventions differed according to BMI: BMI > 30.0 kg/m^2^: 330 kcal/d for 4 weeks; Followed by 1000 kcal/d low-fat diet for 6 months. BMI 25.0–30.0 kg/m^2^: 1000 kcal low-fat diet for 7 months.	Anthropometric measures, insulin, and glucose.	Compared to baseline: ↓ body weight (pre: 91.9 ± 14.5, post: 85.0 ± 13.2 kg; *p* < 0.001); insulin (median (range) mU/L: 9.1 (0.1–31.2) to 4.9 (0.1–19.6); *p* < 0.05); insulin response to oral glucose (426 (46–1113) to 141 (47–918) mU/L; *p* < 0.05). Compared to participants who did not lose >5% of their starting body weight, participants who lost >5% of their starting body weight: ↓ fasting (*p* = 0.018) and glucose-stimulated insulin (*p* < 0.03).	Stratified by those who lost >5% of initial body weight vs. <5%.	Small sample size, no control group, and high attrition rate (41%).
Tolino and colleagues (2005) [64]	Intervention	A total of 144 hirsute individuals with PCOS (BMI > 25.0 kg/m^2^), diagnosed by polycystic ovaries on ultrasound and elevated LH or testosterone.	Interventions differed according to BMI:BMI > 30.0 kg/m^2^: 500 kcal/d for 4 weeks; followed by 1000 kcal/d low-fat diet for 6 months. BMI 25.0–30.0 kg/m^2^: 1000 kcal low-fat diet for 7 months.	Anthropometric measures, glucose, and insulin.	Compared to baseline: ↓ body weight (92 to 86 kg; *p* < 0.001) and insulin (12.3 to 3.4 mU/L; *p* = 0.018). Compared to participants who did not lose >5% of their starting body weight, participants who lost >5% of starting body weight: ↓ insulin response to oral glucose (427 to 142 mU/L; *p* < 0.05), fasting insulin, (12.3 to 3.4 mU/L; *p* = 0.018), and glucose-stimulated insulin (427 to 142 mU/L; *p* < 0.03).	Stratified by those who lost >5% of initial body weight vs. <5%.	No control group and high attrition rate (41%).
Andersen and colleagues (1995) [65]	Single-arm intervention	Nine individuals with PCOS (BMI > 30.0 kg/m^2^), ages 22–39 years, diagnosed by polycystic ovaries on ultrasound with oligomenorrhea/amenorrhea, hirsutism, elevated LH, or hyperandrogenemia.	Two-phase intervention 421 kcal/d for 4 weeks; 1000–15,000 kcal/d for 20 weeks.	Anthropometric measures, lipid profile, and insulin sensitivity.	After 4 weeks, compared to baseline: ↓ BMI (−8%; *p* < 0.01), body fat (−13%; *p* < 0.01), TC e (−29%; *p* = 0.001), TG f (−31%; *p* < 0.05), glucose (−6%; *p* < 0.05), and insulin (−20%; *p* < 0.05); ↑ insulin sensitivity (93%; *p* < 0.05). After 24 weeks, compared to baseline: No difference in body weight, TG, or TC; ↑ insulin sensitivity (86%; *p* < 0.05).		Small sample size, no control group, and high attrition rate (33%).
Magagnini and colleagues (2022) [66]	Retrospective chart review of 3-month intervention	A total of 25 individuals with PCOS (BMI: 30–34.9 kg/m^2^), ages > 18 years, diagnosed by Rotterdam criteria.	Three-phase intervention, each for 4 weeks: Very low-calorie ketogenic diet (600–800 kcal/d); Low-calorie diet (1200–1500 kcal/d); Maintenance stage (1500–200 kcal/d).	Anthropometric measures, lipid profile, glucose, and insulin.	At 3 months, compared to baseline: ↓ WC g (*p* < 0.05), BMI (*p* < 0.05), and HOMA-IR (*p* < 0.05).		Limited diversity and did not discuss dietary adherence.
Other Energy-Restricted Diets							
Holte and colleagues (1995) [67]	Non-randomized control intervention	A total of 13 individuals with PCOS (BMI > 30.0 kg/m^2^) who received the intervention; 21 individuals with PCOS and 23 controls did not receive intervention (BMI: 23–35 kg/m^2^). PCOS was diagnosed by anovulatory menstrual cycle and polycystic ovaries on ultrasound.	Intervention with dietitian (n = 7) or Weight Watchers (n = 7).	Anthropometric measures, glucose, and insulin.	Compared to control group, PCOS intervention: ↓ body weight (−12.4 kg ± 4.7; *p* < 0.0001), WC (pre: 101(95–108) cm, post: 91 (85–98) cm; *p* < 0.0001), and HC ^h^ (pre: 112 (108–116), post: 103 (98–109) cm; *p* < 0.0001); ↓ insulin (pre: 14.8 (8.3–26.3) mU/L, post: 7.6 (5.1–11.5) mU/L; *p* < 0.05) and insulin sensitivity index (pre: 3.2 (2.1–4.3) post: 6.6 (5.0–8.2); *p* < 0.0001).	BMI-matched controls	Different dietary interventions administered but not analyzed separately; no discussion of the intervention or the demographic characteristics.
Moran and colleagues (2007) [68,69]	Intervention	A total of 18 individuals with PCOS and 19 controls (BMI > 25.0 kg/m^2^), PCOS diagnosed by Rotterdam criteria.	Energy restricted diet w/meal replacement beverage for two meals/d, for 8 weeks.	Anthropometric measures, CRP ^j^, lipid profile, glucose, and HOMA-IR.	Compared to baseline, both groups: ↓ body weight (−4.3 ± 3.8 kg), WC (PCOS: −6.1 ± 6.0 cm; non-PCOS: −7.2 ± 4.8 cm), TFM k (PCOS: −2.7 ± 2.5 kg; non-PCOS: −3.2 ± 2.6 cm), TFFM l (PCOS: −1.2 ± 1.6 kg, non-PCOS: −1.0 ± 2.4 kg), and TG (PCOS: −0.3 ± 1.5 mmol/L; non-PCOS −0.2 ± 0.8 mmol/L), with no sig. difference between groups; No sig. change in TC, LDL-C, m HDL-C, n and glucose. Compared to control group, PCOS: ↑ insulin resistance after 8 weeks (*p* = 0.026).	Matched for BMI and smoking status.	Lack of diversity and small sample size.
Moini and colleagues (2019) [70]	Intervention	A total of 90 individuals with PCOS (BMI ≥ 28.0 kg/m^2^), ages 18–40 years, diagnosed by Rotterdam criteria.	Individually designed energy-restricted diets (1000 kcal/d energy deficit) for 12 weeks.	Anthropometric measures and insulin; improvements in menstrual cyclicity (responders) vs. no improvements (non-responders).	Compared to baseline: ↓ body weight (responders: −7.5 ± 0.5 kg; non-responders −7.6 ± 0.5 kg), WC (responders: −5.6 ± 0.2 cm; non-responders −5.6 ± 0.1 cm), and insulin (responders: −2.6 ± 0.1 mU/L; non-responders: −2.9 ± 0.3 mU/L), with no sig. different between responders and non-responders.	Stratified by responders and non-responders.	No control group and all participants were infertile.
Soares and colleagues (2016) [71]	Intervention	A total of 22 individuals with PCOS (BMI ≥ 25.0 and <39.0 kg/m^2^), ages 18–35 years, diagnosed by Rotterdam criteria.	Energy-restricted diet (500 kcal/d energy deficit) for 12 weeks.	Anthropometric measures, lipid profile, glucose, and insulin.	Compared to baseline: ↓ BMI (pre: 29.8 ± 6.1 kg/m^2^, post: 28.9 ± 5.8 kg/m^2^; *p* = 0.001), glucose (pre: 79 ± 9.6 mg/dL, post: 74.6 ± 8.7; *p* = 0.027), insulin (pre: 11.3 (8.07–15.07) μIU/mL, post: 5.5 (4.1–7.6) μIU/mL; *p* = 0.001), HOMA-IR (pre: 1.9 (1.3–3.3) mol× μU/L, post: 0.9 (0.7–1.3) mol× μU/L; *p* = 0.001), and LDL-C (pre: 89 (70–104) mg/dL, post: 86 (50–50) mg/dL; *p* = 0.001); No significant change in WC, TG, and HDL-C.		No control group and small sample size.
Macronutrient Distribution Low Carbohydrate							
Douglas and colleagues (2006) [72]	Crossover intervention	A total of 11 individuals with PCOS, diagnosed by 1990 National Institute of Health criteria.	Three eucaloric dietary interventions each for 16 days with 3 week washout: Low CHO ^o^ (43% CHO, 15% protein, 45% fat); ADA ^p^ diet (56% CHO, 16% protein, 31% fat); Enriched MUFA ^q^ diet (55% CHO, 15% protein, 33% fat).	Glucose, insulin, insulin sensitivity, and lipid profile.	Compared to the ADA diet, the low CHO demonstrated the following: ↓ insulin (ADA: 17.5 ± 7.2 μ IU/mL, low CHO: 14.3 ± 8.2 μIU/mL; *p* = 0.03) and TC (ADA: 165.4 ± 54.1 mg/dL, low CHO: 148.6 ± 47.1 mg/dL; *p* = 0.01).		Small sample size, potential effect of diet treatment order, and short intervention period.
Gower and colleagues (2013) [73]	Crossover intervention	A total of 30 individuals with PCOS (BMI < 45.0 kg/m^2^), diagnosed by 1990 National Institute of Health criteria.	Two eucaloric dietary interventions each for 8 weeks with 4 week washout: Reduced CHO diet (41% CHO, 19% protein, and 40% fat); Standard diet (55% CHO, 18%protein, and 27% fat).	Anthropometric measures, lipid profile, and insulin.	Compared to baseline, both diets demonstrated the following: ↓ body weight (standard diet: −1.30 kg, reduced CHO: −1.66 kg, *p* = 0.558). Compared to the standard diet, the reduced CHO diet demonstrated the following: ↓ HOMA-IR (pre: 2.4 ± 2.1, post: 1.7 ± 1.4; *p* < 0.001), insulin (pre: 58.8 ± 47.5 pM, post: 43.2 ± 32.4 pM, *p* < 0.001), glucose (pre: 5.30 ± 0.47 mM, post: 5.04 ± 0.47; *p* < 0.01), TC (pre: 4.75 ± 0.84 mM, post: 4.22 ± 0.65; *p* < 0.001), LDL-C (pre: 2.97 ± 0.84 mM, post: 2.56 ± 0.66 mM; *p* < 0.01), and HDL-C (reduced CHO: 1.38 ± 0.39 mM, post: 1.27 ± 0.39; *p* < 0.05); ↑ insulin sensitivity index (pre: 6.4 ± 4.2, post: 7.6 ± 5.0; *p* < 0.05). Compared to the reduced CHO diet, the standard diet showed the following: ↓ HDL-C (pre: 1.40 ± 0.40, post: 1.27 ± 0.39 mM; *p* < 0.01); ↑ cholesterol-to-HDL-C ratio (pre: 3.54 ± 0.95, post: 3.88 ± 1.16; *p* < 0.05).		Relatively small sample size.
Perelman and colleagues (2017) [74]	Randomized crossover intervention	$\mathrm{Six}\;\mathrm{pre}-\mathrm{menopausal}\;\mathrm{individuals}\;(\mathrm{BMI}\;>\;25.0\;\mathrm{kg}/m^{2})\;\mathrm{with}\;\mathrm{PCOS},\;\mathrm{ages}\;\leq$ 40 years, diagnosed by 1990 National Institute of Health criteria.	Two eucaloric dietary interventions each for 3 weeks, with 2 week washout: Low-CHO/fat-enriched diet (40% CHO, 15% protein, 45% fat); Higher CHO diet (60% CHO, 15% protein, 25% fat).	Anthropometric measures, insulin, glucose, and lipid profile.	Compared to the higher CHO diet, the low-CHO/fat enriched diet demonstrated the following: ↓ AUCins ^i^ (450 ± 140 versus 644 ± 174 mU/mL 8 h; *p* = 0.02) and LDL-C (−12 ± 60 mg/dL; *p* < 0.05).		Limited diversity, small sample size, short study duration
Protein Modification							
Karamali and colleagues (2018) [75]	Randomized controlled trial	60 individuals with PCOS, ages 18–40 years, PCOS diagnosed by Rotterdam criteria	Textured soy protein diet: 0.8 g protein/kg body weight (35% animal protein, 35% textured soy protein, 30% vegetable proteins) for 8 weeks or Control diet: Similar diet, without textured soy protein (70% animal proteins, 30% vegetable proteins) for 8 weeks.	Anthropometric measures, insulin, glucose, lipid profile, CRP	Compared to the control diet, the textured soy protein diet showed the following: ↓ body weight (−0.7 ± 1.5 versus +0.1 ± 1.2 kg; *p* = 0.02), BMI (−0.3 ± 0.6 versus +0.1 ± 0.5 kg/m^2^; *p* = 0.02), WC (−1.2 ± 1.7 versus +0.1 ± 1.3 cm; *p* = 0.002), HC (−1.6 ± 1.8 versus +0.2 ± 1.4 cm; *p* < 0.001), insulin (−15.0 ± 18.0 versus +4.8 ± 18.6 pmol L^−1^; *p* < 0.001), HOMA-IR (−0.6 ± 0.6 versus +0.2 ± 0.7; *p* < 0.001), TG (−0.1 ± 0.4 versus −0.2 ± 0.3 mmol L^−1^; *p* = 0.01), and VLDL-C ^r^ (−0.1 ± 0.1 versus +0.0 ± 0.1 mmol L^−1^; *p* = 0.01); ↑ QUICKI ^s^ (+0.01 ± 0.01 versus −0.00 ± 0.02; *p* = 0.01).	BMI, age, PCOS phenotype	Did not include a group that was not on metformin
Sorensen and colleagues (2012) [76]	Parallel group controlled trial	A total of 57 individuals with PCOS, diagnosed by Rotterdam criteria.	High-protein diet: Ad libitum energy intake (>40% protein, 30% fat, <30% CHO) for 6 months; or Standard-protein diet: Ad libitum energy intake (<15% protein, 30% fat, >55% CHO) for 6 months.	Anthropometric measures, lipid profile, and glucose.	Compared to the standard-protein diet, the high-protein diet showed the following: ↓ body weight (high protein: 71.4 (63.3, 84.9) kg, standard protein: 75.4 (68.1, 82.8) kg; *p* = 0.002), body fat (high protein: 27.2 (18.5, 36.2) kg, standard protein: 29.4 (23.2, 35.6) kg; *p* = 0.002), WC high protein: 88.3 (78.5, 98.0) cm, standard protein: 89.7 (83.6, 95.7) cm; *p* = 0.04), and glucose (high protein: 5.2 (5.0, 5.3) mmol/L, standard protein: 5.4 (5.3, 5.6) mmol/L; *p* = 0.03).	Intention to treat analyses; adjusted analysis for body weight loss.	High attrition rate (53%).
High-Saturated Fat, Starch Avoidant Diet							
Hays and colleagues (2003) [77]	Single-arm study	A total of 15 individuals with PCOS (BMI > 30.0 kg/m^2^), ages 21–43 years.	High-saturated fat and starch avoidant diet for 24 weeks.	Lipid profile, CRP, glucose, and insulin.	Compared to baseline: ↓ BMI (pre: 36.1 ± 9.7, post: 32.4 ± 8.9; *p* < 0.001), % total body weight (−14.3 ± 20.3%; *p* = 0.008), and insulin (pre: 24.2 ± 11.8 mg/dL, post: 12.2 ± 5.0 mg/dL; *p* = 0.005).		No control group and no description of participant demographics or diagnostic criteria.
Dietary Pattern Low Glycemic Index							
Marsh and colleagues (2010) [78]	Intervention	A total of 96 individuals with PCOS (BMI ≥ 25.0 kg/m^2^), ages 18–40 years, PCOS diagnosed by self-reported Rotterdam criteria.	Low-fat, low GI ^t^ diet: Ad libitum intake (50% CHO, 23% protein, 27% fat, GI 40%) until loss of 7% baseline body weight: or Low-fat, conventional healthy diet: Ad libitum intake (50% CHO, 23% protein, 27% fat, GI: 59%) until loss of 7% baseline body weight.	Anthropometric measures, lipid profile, insulin, and CRP.	Compared to the low-fat, conventional healthy diet, the low GI diet showed the following: ↑ insulin sensitivity index derived from oral glucose tolerance test (low GI 2.2 ± 0.7, conventional 0.7 ± 0.6; *p* = 0.03); ↑ insulin sensitivity in those on metformin (*p* = 0.048).	Body weight loss and metformin use.	High attrition rate (49%) and non-randomized.
Barr and colleagues (2013) [79]	Non-randomized trial	A total of 26 pre-menopausal individuals with PCOS, ages ≥ 18 years, diagnosed by self-report with general practitioner confirmation.	Three dietary phases, each for 12 weeks Habitual diet control phase; Isocaloric low GI diet; No intervention follow-up.	Anthropometric measures, blood pressure, glucose, insulin, and lipid profile.	Compared to baseline, at 24 weeks: ↓ HDL-C (1.7 ± 0.5 mmol/L to 1.6 ± 0.4 mmol/L; *p* = 0.05); ↑ insulin sensitivity (72.8 ± 32.0% vs. 61.1 ± 24.9%; *p* = 0.03); No sig. changes in glucose, other lipids, body weight, or WC. Compared to baseline, at 36 weeks: No sig. changes. Compared to 24 weeks, at 36 weeks: ↑ GI intake (*p* < 0.001).		High attrition rate (30%) in follow up period, small sample size, and no comparison group.
Panico and colleagues (2014) [80]	Randomized crossover design	Seven individuals with PCOS, diagnosed by Rotterdam criteria.	Two isocaloric dietary interventions each for 3 months: Moderately low GL ^u^; Moderately high GL.	Insulin sensitivity, glucose, and lipid profile.	Compared to baseline at 3 months of the low GL diet: ↓ HOMA-IR 2 h after breakfast (pre: 9.82 ± 4.97, post: 5.38 ± 4.72; p< 0.036), glucose 2 h after breakfast (pre: 89.7 ± 9.0, post: 78.6 ± 3.7 mg/dL; p< 0.011), and insulin 2 h after breakfast (pre: 44.9 ± 24.0, post: 18.4 ±10.3 μU/mL; *p* = 0.019)	Age and socioeconomic background.	Small sample size and not clear if there was a washout period.
Low Starch, Low Dairy							
Pohlmeier and colleagues (2014) [81]	Single-arm intervention	10 individuals with PCOS (BMI 25.0–45.0 kg/m^2^), ages 18–45 years, PCOS diagnosed by Rotterdam criteria	Low-starch/low-dairy diet for 8 weeks	Anthropometric measures, insulin, glucose	Compared to baseline: $↓\;\mathrm{body}\;\mathrm{weight}\;(-8.1\;\pm \;1.8\;\mathrm{kg};\;p<\;0.05),\;\mathrm{BMI}\;(-3.0\;\pm \;0.6\;\mathrm{kg}/m^{2};\;p<\;0.05),\;\mathrm{WC}\;(-3.0\;\pm \;1.3\;\mathrm{in};\;p<\;0.05),\;\mathrm{HC}\;(-2.5\;\pm \;1.3\;\mathrm{in};\;p<\;0.05),\;\mathrm{body}\;\mathrm{fat}\;(-6.6\;\pm \;3.8\;\mathrm{kg};\;p<\;0.05),\;\mathrm{BF}\%\;v^{\;}(-2.3\;\pm \;2.5\%;\;p<\;0.05),\;\mathrm{insulin}\;(-20\;\pm \;9\;\mu$g/mL; *p* < 0.05), and 2 h insulin (−139 ± 230 μg/mL; *p* < 0.05).		Small sample size, no control group, and lack of pre-intervention dietary intake.
Ketogenic Mediterranean							
Paoli and colleagues (2020) [82]	Single-arm intervention	A total of 14 individuals with PCOS (BMI ≥ 25.0 kg/m^2^), ages 18–45 years, diagnosed by Rotterdam criteria.	Ketogenic Mediterranean diet for 12 weeks.	Anthropometric measurements, glucose, insulin, and lipid profile,	Compared to baseline: $↓\;\mathrm{body}\;\mathrm{weight}\;(\mathrm{pre}:\;81.2\;\pm \;8.4\;\mathrm{kg},\;\mathrm{post}:\;71.8\;\pm \;6.7\;\mathrm{kg};\;p<\;0.001),\;\mathrm{BMI}\;(\mathrm{pre}:\;28.8\;\pm \;2.1,\;\mathrm{post}:\;25.5\;\pm \;1.7\;\mathrm{kg}/m^{2};\;p<\;0.001),\;\mathrm{body}\;\mathrm{fat}\;(\mathrm{pre}:\;28.0\;\pm \;5.1\;\mathrm{kg},\;\mathrm{post}:\;19.7\;\pm \;3.7\;\mathrm{kg};\;p<\;0.001),\;\mathrm{LBM}\;w^{\;}(\mathrm{pre}:\;53.2\;\pm \;5.0\;\mathrm{kg},\;\mathrm{post}:\;52.1\;\pm \;4.6\;\mathrm{kg};\;p=\;0.021),\;\mathrm{VAT},{\;}^{x}\;(\mathrm{pre}:\;1750\;\pm \;182\;\mathrm{grams},\;\mathrm{post}:\;1110\;\pm \;189;\;p<\;0.001),\;\mathrm{WC}\;(\mathrm{pre}:\;101\;\pm \;5,\;\mathrm{post}:\;97\;\pm \;4\;\mathrm{cm};\;p=\;0.0015),\;\mathrm{glucose}\;(\mathrm{pre}:\;5.1\;\pm \;0.3\;\mathrm{mmol}/L,\;\mathrm{post}:\;4.6\;\pm \;0.2;\;p<\;0.001),\;\mathrm{insulin}\;(\mathrm{pre}:\;12.6\;\pm \;0.5\;\mu$U/mL, post: 11.3 ± 0.6 μU/mL; *p* < 0.001), HOMA-IR (pre: 2.9 ± 0.2, post: 2.3 ± 0.1; *p* < 0.001), TG (pre: 2.3 ± 0.4 mmol/L, post: 1.9 ± 0.3 mmol/L; *p* < 0.001), TC (pre: 5.4 ± 0.4 mmol/L, post: 4.7 ± 0.3 mmol/L; *p* < 0.001), and LDL-C (pre: 3.1 ± 0.6 mmol/L, post: 2.3 ± 0.2 mmol/L; *p* < 0.001); ↑ HDL-C (pre: 1.8 ± 0.41 mmol/L, post: 2.0 ± 0.4 mmol/L; *p* < 0.001).		Described as both a eucaloric and low-calorie intervention, small sample size, a lack of control group, and a lack of demographic information.
Eating Behavior Meal Timings/Frequency							
Li and colleagues (2021) [83]	Non-randomized intervention	A total of 15 individuals with PCOS (BMI ≥ 24.0 kg/m^2^), ages 18–40 years, diagnosed by Rotterdam criteria.	Ad libitum time-restricted feeding (8 am−4 pm) for 5 weeks.	Anthropometric measures, insulin, CRP, and lipid profile.	Compared to baseline: ↓ BMI (pre: 29.8 ± 4.3 kg/m^2^, post: 28.6 ± 4.4 kg/m^2^; *p* < 0.001), body fat (pre: 35.3 ± 10.0 kg, post: 32.9 ± 9.9 kg; *p* < 0.001), BF% (pre: 40.1 (39.8–47.6), post: 39.7 (38.4–46.0); *p* = 0.001), VFA ^y^ (pre: 165 ± 39 cm^2^, post: 155 ± 41 cm^2^; *p* = 0.015), HOMA-IR (pre: 3.5 (2.9–5.6), post: 2.7 (2.3–3.9); *p* = 0.025), AUCIns/AUCGlu ^z^ ratio (pre: 16.5 ± 5.9, post: 11.8 ± 4.8; *p* = 0.001), and CRP (pre: 4.9 ± 3.2, post: 2.9 ± 1.6 mg/L; *p* = 0.040); No sig. decrease in glucose, AUCGlu, TG, TC, or LDL-C.		No control group, small sample size, and short duration.
Jakubowicz and colleagues (2013) [84]	Randomized parallel-arm intervention	A total of 60 individuals with PCOS (BMI < 24.9 kg/m^2^), ages 25–39 years, diagnosed by Rotterdam criteria.	High-calorie breakfast diet (980 kcal) or high-calorie dinner (980 kcal) diet for 12 weeks.	Anthropometric measures, blood pressure, glucose, and insulin.	Compared to high-calorie dinner group, the high-calorie breakfast group showed the following: $↓\;\mathrm{HOMA}-\mathrm{IR}\;(-56\%;\;p<\;0.001),\;\mathrm{and}\;\mathrm{HOMA}-B{\;}^{\mathrm{aa}}\;(-35\%;\;p=\;0.001),\;\mathrm{glucose}\;(\mathrm{pre}:\;89\;\pm \;1,\;\mathrm{post}:\;82\;\pm \;1\;\mathrm{mg}/\mathrm{dL};\;p\;<\;0.001),\;\mathrm{insulin}\;(\mathrm{pre}:\;14.3\;\pm \;0.9,\;\mathrm{post}:\;6.7\;\pm \;0.3\;\mu$-IU/mL; *p* < 0.001), AUCglu (pre: 17,429 ± 155 to 13,918 ± 81 mg/dL post; *p* < 0.001), and AUCins (pre: 7361 ± 156 to 3774 ± 94 μ-IU/mL; *p* < 0.001).		No control group.
Papakonstantinou and colleagues (2016) [85]	Randomized crossover study	A total of 40 individuals with PCOS, diagnosed by Rotterdam criteria.	Two eucaloric dietary patterns each for 12 weeks: 3 meals/d; 6 meals/d.	Anthropometric measures, OGTT, glucose, insulin, and lipid profile.	Compared to baseline, both groups showed the following: ↓ WC with no sig. difference between groups (*p* = 0.163). Compared to 3 meals/d group, 6 meals/d group showed the following: ↓ insulin (pre: 96 ± 12, post: 92 ± 10 pmol/l; *p* = 0.03); ↑ post-OGTT insulin sensitivity (pre: 4.4 ± 0.4, post: 5.3 ± 0.7; *p* = 0.02).	Adjustments for family history of diabetes.	Did not include a washout period in between interventions.
Asemi and colleagues (2015) [86]	Quasi-experimental trial	A total of 27 individuals with PCOS, ages 18–40 years, diagnosed by Rotterdam criteria.	Ramadan fasting (mean fasting period of 16.5 h/d) for 30 days.	Body weight, glucose, insulin sensitivity, lipid profile, and CRP.	Compared to baseline: No sig. difference in body weight, BMI, glucose, or lipid profiles.		No control or comparison group.
Modification of Advanced Glycation End Products							
Tantalaki and colleagues (2014) [87]	Intervention	A total of 34 individuals with PCOS, ages 18–40 years, diagnosed by 1990 National Institute of Health criteria.	Three dietary phases, each 2 months Hypocaloric diet w/ad libitum AGEs ^bb^ content; Isocaloric diet with high AGEs; Isocaloric diet with low AGEs.	Anthropometric measures glucose, and insulin.	Compared to baseline, all groups showed the following: ↓ BMI (*p* < 0.05) Compared to hypocaloric diet w/ad libitum AGEs, high AGEs diet showed the following: ↑ insulin (hypo: 10.6 ± 5.2 μIU/mL, high AGEs: 13.6 ± 6.3 μIU/mL; *p* < 0.05); HOMA-IR (hypo: 2.3 ± 1.2, post: 2.9 ± 1.4; *p* < 0.05). Compared to isocaloric diet with high AGES, the isocaloric, low AGES diet showed the following: ↓ glucose (high AGEs: 87 ± 6 mg/dL, low AGEs: 83 ± 8; p< 0.05), insulin (high AGES: 13.6 ± 6.3 μIU/mL, low AGEs 9.2 ± 2.8 μIU/mL; *p* < 0.05), and HOMA-IR (high AGEs: 2.9 ± 1.4, low AGEs: 1.9 ± 0.6; *p* < 0.05).		Lack of washout period; high attrition rate (32%).
Energy Restriction and Macronutrient Distribution Energy Restricted Diet with Protein Modification							
Kasim-Karakas and colleagues (2009) [88]	Randomized single-blind trial	A total of 33 individuals with PCOS (BMI: 25.0–40.0 kg/m^2^), ages 18–45 years, diagnosed by 1990 National Institute of Health criteria.	Energy restricted with powdered protein supplement: 700 kcal/d energy deficit; 240 kcal, whey protein isolate for 2 months; or Energy restricted with simple sugar supplement: 700 kcal/d energy deficit; 240 kcal, glucose + maltose for 2 months.	Anthropometric measures, lipid profile, glucose, and insulin.	Compared to the energy-restricted diet with simple sugar supplement, the energy-restricted diet with protein supplement group showed the following: ↓ body weight (−3.4± 0.8 kg; *p* < 0.03), BMI (pre: 38.9 ± 1.6 g/m^2^, post: 37.1 ± 1.8 kg/m^2^; *p* < 0.03), body fat (−3.1 ± 0.9 kg; *p* < 0.03), TC (pre: 201 ± 8 mg/dL, post: 168 ±10 mg/dL; *p* < 0.006), and HDL-C (pre: 38 ± 2 mg/dL, post: 34 ± 2 mg/dL; *p* < 0.04).		No control group.
Moran and colleagues (2003) [89,90]	Randomized intervention	A total of 45 individuals with PCOS (BMI >25.0 kg/m^2^), diagnosed by 1990 National Institute of Health criteria.	Low-protein diet: 55% CHO, 15% protein, 30% fat; or High-protein diet: 40% CHO, 30% protein, 30% fat. Energy restricted for 12 weeks, then weight maintenance for 4 weeks.	Anthropometric measures, glucose, insulin, and lipid profile.	Compared to baseline, both groups showed the following: ↓ body weight (−7.7 ± 0.7 kg), body fat (−14.4%; *p* < 0.001), LBM (−3.4%; *p* < 0.001), abdominal fat mass (−12.5%; *p* < 0.001), TC −8.8%; *p* < 0.001), TG (−12.5%; *p* < 0.001), LDL-C (−9.8%; *p* < 0.001), insulin (−20%; *p* = 0.001), and HOMA-IR (−9%; *p* = 0.001). Compared to the high-protein diet, the low-protein diet showed the following: ↓ HDL-C (−10%; *p* < 0.001). Compared to the low-protein diet, the high-protein diet showed the following: ↓ TC/HDL-C (−12.5%; *p* < 0.003).	Participants stratified on body weight, age, and desire to conceive.	No control group; high attrition rate (38%).
Toscani and colleagues (2011) [91] ^†^	Single-blind randomized control trial	A total of 18 individuals with PCOS and 22 controls (BMI 18.5–39.9 kg/m^2^), ages 14–35 years; PCOS diagnosed by 2006 Androgen Excess Society criteria.	Energy restricted high protein: 30% protein, 40% CHO, 30% fat for 2 months; or Energy restricted normal protein: 15% protein, 55% CHO, 30% fat) for 2 months. 20–25 kcal/kg/d.	Anthropometric measurements, glucose, insulin, and lipid profile.	Compared to baseline, both groups showed the following: ↓ body weight (*p* < 0.001), BMI, (*p* < 0.001), WC (*p* < 0.001), and BF%, (*p* < 0.001), with no sig. differences between groups.	BMI-matched controls.	Limited diversity.
Stamets and colleagues (2004) [92]	Randomized pilot trial	A total of 35 individuals with PCOS (BMI ≥ 25.0 kg/m^2^), ages 21–37 years, diagnosed by 1990 National Institute of Health criteria.	Energy restricted high protein: 30% protein, 40% CHO, and 30% fat for 4 weeks; or Energy restricted high CHO: 15% protein, 55% CHO, and 30% fat for 4 weeks. 1000 kcal/d deficit.	Anthropometric measures, glucose, and insulin.	Compared to baseline, both diets showed the following: ↓ body weight (high protein: −3.7 ± 1.9 kg, high CHO: −4.4 ± 1.5 kg); no sig. difference between groups.	Combined the two groups to analyze the effect of hypocaloric diet on metabolic markers.	7% attrition in first week d/t inability to tolerate intervention.
Energy Restriction with Carbohydrate/Fat Restriction							
Moran and colleagues (2006) [93]	Randomized intervention	A total of 43 individuals with PCOS (BMI >25.0 kg/m^2^), diagnosed by Rotterdam criteria.	Two-phase dietary intervention: 8 weeks energy restriction with 2 meals/d replaced with meal replacement beverage; 24 weeks of body weight maintenance with CHO restriction (<120 g/CHO/d) counting or fat restriction (<50 g/fat/d).	Anthropometric measures, glucose, insulin sensitivity, and lipid profile.	Compared to baseline, after energy restriction: ↓ body weight (−5.6 ± 2.4 kg; *p* < 0.001), WC (6.1 ± 0.4%; *p* < 0.001), TFFM (−2.5 ± 0.5%; *p* < 0.001), TFM (−12.3 ± 1.4; *p* < 0.001), TC (−2.6 ± 3.3%; *p* < 0.001), TG (−12.9 ± 5.2%; *p* = 0.005), LDL-C (−12.1 ± 3.3%; *p* < 0.001), HDL-C (−9.5 ± 3.5%; *p* < 0.001), CRP (−9.6 ± 8.2%; *p* = 0.018), glucose (−1.8 ± 1.0%; *p* = 0.02), and SBP ^cc^ (−6.6 ± 1.4%; *p* < 0.001). Compared to 8 weeks, at 32 weeks: ↑ body weight (+2.5 ± 0.7 kg; *p* = 0.028). Compared to baseline, at 32 weeks: ↓ body weight (net weight loss of 3.2 ± 0.7 kg), glucose (net decrease of 2.9 ± 1.2%; *p* = 0.046), SBP (net decrease of 6.6 ± 2.2%; *p* = 0.002), insulin (net decrease of 3.5 ± 1.6 mU/L; *p* = 0.044), and HOMA-IR (net decrease of 0.9 ± 0.4; *p* = 0.033).	Stratified for equal distribution of age, BMI, smoking status, and use of oral contraceptive.	High attrition rate (32% in phase 2), no control group, and lack of diversity.
Energy Restriction and Dietary Pattern Energy-Restricted Dietary Approaches to Stop Hypertension							
Asemi and colleagues (2014) [94]	Randomized controlled trial	A total of 48 individuals with PCOS (BMI ≥ 25.0 kg/m^2^), ages 18–40 years, diagnosed by Rotterdam criteria.	Energy-restricted DASH: ^dd^ 350–700 kcal/d deficit for 8 weeks; or Energy-restricted control: 350–700 kcal/d deficit for 8 weeks.	Anthropometric measures, glucose, and lipid profiles.	Compared to control diet, the DASH diet showed the following:↓ body weight (DASH: −4.4 ± 2.7, control: −1.5 ± 2.6 kg; *p* < 0.001), BMI (DASH: −1.7 ± 1.1, control: 0.6 ± 0.9 kg/m^2^; *p* < 0.001), TG (DASH: −10.0 ± 22.3 mg/dL, control: 19.2 ± 42.8 mg/dL; *p* = 0.005), VLDL-C (DASH: −2.0 ± 4.5 mg/dL, control: 3.9 ± 8.6; *p* = 0.005), and insulin (DASH −1.88 μIU/mL, control: 2.89 μIU/mL; *p* = 0.03).	BMI; age.	
Foroozanfard and colleagues (2017) [95]	Randomized controlled trial	A total of 60 individuals with PCOS (BMI > 25.0 kg/m^2^), ages 18–40 years, diagnosed by Rotterdam criteria.	Energy-restricted DASH: 350–700 kcal/d deficit for 12 weeks; or Energy-restricted control: 350–700 kcal/d deficit for 12 weeks.	Insulin sensitivity; glucose.	Compared to control diet, the DASH diet showed the following: ↓ body weight (DASH: −4.3 ± 1.4 kg, control: −3.2 ± 1.9 kg; *p* = 0.01), BMI (DASH: −1.6 ± 0.5 kg/m^2^, control: −1.2 ± 0.7 kg/m^2^; *p* = 0.02), insulin (DASH: −25.2 ± 51.0 pmol/L, control: −1.2 ± 28.8 pmol/L; *p* = 0.02), and HOMA-IR (DASH: −0.9 ± 2.0, control: −0.1 ± 1.0; *p* = 0.02); ↓ QUICKI (DASH: 0.01 ± 0.03, control: −0.00 ± 0.01; *p* = 0.02).	BMI; age.	Did not include lipids or other markers of cardiometabolic risk.
Energy-Restricted Low-Glycemic Index Diet							
Shishehgar and colleagues (2019) [96]	Intervention study	A total of 33 individuals with PCOS and 40 controls (BMI > 25 kg/m^2^), ages 18–40 years; PCOS diagnosed by Rotterdam criteria.	Energy-restricted (500 kcal/d deficit) low-GI index diet for 24 weeks.	Body weight, glucose, insulin, and blood pressure.	Compared to baseline, both groups showed the following: ↓ body weight (PCOS: −6.7 ± 0.6 kg, controls: −6.2 ± 0.5 kg), BMI (PCOS: −2.6 ± 0.2 kg/m^2^, controls: −2.5 ± 0.2 kg/m^2^), and insulin (PCOS: −4.8 ± 1.6 mU/L, controls: −3.8 ± 1.3 mU/L) with no sig. differences between groups. Compared to control group, PCOS group showed the following: ↓ DBP ^ee^ (−1.2 ± 0.7 mmHg; *p* = 0.03).	Age and BMI; non-PCOS controls.	Body composition and lipid profiles not assessed.
Szczuko and colleagues (2018) [97,98] ^‡^	Intervention	A total of 24 individuals with PCOS, ages 17–38 years, diagnosed by Rotterdam criteria.	Energy-restricted (600 kcal/d deficit) low GI diet for 3 months,	Anthropometric measures, glucose, insulin, and lipid profile.	Compared to baseline: ↓ body weight (pre: 79.1 ± 14.6 kg, post: 73.0 ± 10.2 kg; *p* < 0.05), BMI (pre: 28.8 ± 5.6 kg/m^2^, post: 26.6 ± 7.3; *p* < 0.05), WC (pre: 97.9 ± 12.1 cm, post: 90.4 ± 12.7; *p* < 0.05), WHR ^ff^ (pre: 0.9 ± 0.1, post: 0.9 ± 0.1; *p* < 0.05), body fat (pre: 30.7 ± 9.7 kg, post: 26.6 ± 8.1 kg; *p* < 0.05), TG (pre: 133 ± 40 mg/dL, post: 71 ± 30 mg/dL; *p* < 0.05), LDL-C (pre: 129 ± 30 mg/dL, post: 98 ± 16 mg/dL; *p* < 0.01), and TC (pre: 225 ± 25 mg/dL, post: 172 ± 22 mg/dL; *p* < 0.05).		No control group; no discussion of how compliance was measured.
Mehrabani and colleagues (2012) [99]	Randomized controlled trial	A total of 60 individuals with PCOS (BMI >25.0 and <38.0 kg/m^2^), ages 20–40 years, diagnosed by 1990 National Institute of Health criteria.	Low–medium GI energy-restricted diet: 500–1000 kcal/d deficit (40% low and medium GI CHO, 30% protein, 30% fat) for 12 weeks; or Energy-restricted diet 500–1000 kcal/d deficit (55% CHO, 15% protein, 30% fat) for 12 weeks.	Body weight, glucose, insulin, CRP, and lipid profile.	Compared to baseline, both diets showed the following: ↓ body weight (low–medium GI: −4.1 ± 0.6%, energy restricted: −3.3 ± 0.6%) and LDL-C (low–medium GI: −41.3 ±4.3 mg/dL, energy restricted: −38.5 ± 4.9 mg/dL), with no sig. difference between groups. Compared to the energy-restricted diet, the low–medium GI energy-restricted diet showed the following: ↓ WC (*p* < 0.05), CRP (−0.9 ± 0.4 mg/mL; *p* < 0.05), insulin −3.6 ± 0.7 mU/mL; *p* < 0.05), and HOMA-IR (−0.8 ± 0.2; *p* < 0.05).	Stratified for age and BMI.	

↑: Increased; ↓: Decreased; ^†^ Included participants aged 14–35 years, mean age was 22.72 ±  5.68 years; ^‡^ included participants aged 17–38 years; ^a^ PCOS: Polycystic Ovary Syndrome; ^b^ BMI: Body Mass Index; ^c^ OGTT: Oral Glucose Tolerance Test; ^d^ HOMA-IR: Homeostatic Model Assessment of Insulin Resistance; ^e^ TC: Total Cholesterol; ^f^ TG: Triglycerides; ^g^ WC: Waist Circumference; ^h^ HC: Hip Circumference; ^i^ AUCins: Area Under the Curve Insulin; ^j^ CRP: C-Reactive Protein; ^k^ TFM: Total Fat Mass; ^l^ TFFM: Total Fat-Free Mass; ^m^ LDL-C: Low-Density Lipoprotein Cholesterol; ^n^ HDL-C: High-Density Lipoprotein Cholesterol; ^o^ CHO: Carbohydrate; ^p^ ADA: American Diabetes Association; ^q^ MUFA: Monounsaturated Fatty Acids; ^r^ VLDL-C: Very Low-Density Lipoprotein Cholesterol; ^s^ QUICKI: Quantitative Insulin-Sensitivity Check Index; ^t^ GI: Glycemic Index; ^u^ GL: Glycemic Load; ^v^ BF%: Body Fat Percentage; ^w^ LBM: Lean Body Mass; ^x^ VAT: Visceral Adipose Tissue; ^y^ VFA: Visceral Fat Area; ^z^ AUCglu: Area Under the Curve Glucose; ^aa^ HOMA-B: Homeostasis Model Assessment of Beta Cell Function; ^bb^ AGES: Advanced Glycation End Products; ^cc^ SBP: Systolic Blood Pressure; ^dd^ DASH: Dietary Approaches to Stop Hypertension; ^ee^ DBP: Diastolic Blood Pressure; ^ff^ WHR: Waist/Hip Ratio.

## Data Availability

No new data were created or analyzed in this study. Data sharing is not applicable to this article.
